# The impact of the initial COVID-19 outbreak on young adults’ mental health: a longitudinal study of risk and resilience factors

**DOI:** 10.1038/s41598-022-21053-2

**Published:** 2022-10-05

**Authors:** Anna Wiedemann, Jan Stochl, Sharon A. S. Neufeld, Jessica Fritz, Junaid Bhatti, Roxanne W. Hook, NSPN Consortium, Edward Bullmore, Raymond Dolan, Ian Goodyer, Peter Fonagy, Peter Jones, Michael Moutoussis, Tobias Hauser, Sharon Neufeld, Rafael Romero-Garcia, Michelle St. Clair, Petra Vértes, Kirstie Whitaker, Becky Inkster, Gita Prabhu, Cinly Ooi, Umar Toseeb, Barry Widmer, Junaid Bhatti, Laura Villis, Ayesha Alrumaithi, Sarah Birt, Aislinn Bowler, Kalia Cleridou, Hina Dadabhoy, Emma Davies, Ashlyn Firkins, Sian Granville, Elizabeth Harding, Alexandra Hopkins, Daniel Isaacs, Janchai King, Danae Kokorikou, Christina Maurice, Cleo McIntosh, Jessica Memarzia, Harriet Mills, Ciara O’Donnell, Sara Pantaleone, Jenny Scott, Beatrice Kiddle, Ela Polek, Pasco Fearon, John Suckling, Anne-Laura van Harmelen, Rogier Kievit, Sam Chamberlain, Richard A. I. Bethlehem, Ian M. Goodyer, Raymond J. Dolan, Edward T. Bullmore, Samuel R. Chamberlain, Peter Fonagy, Jesus Perez, Peter B. Jones

**Affiliations:** 1 Department of Psychiatry, University of Cambridge, Douglas House, 18B Trumpington Road, Cambridge, CB2 8AH UK; 2 Cambridgeshire and Peterborough NHS Foundation Trust, Cambridge, UK; 3 National Institute for Health Research, Applied Research Collaboration, East of England, Cambridge, UK; 4 Department of Kinanthropology and Humanities, Charles University, Prague, Czechia; 5 Department of Clinical Psychology, Philipps University of Marburg, Marburg, Germany; 6 Max Planck UCL Centre for Computational Psychiatry and Ageing Research, London, UK; 7 Department of Psychiatry, Faculty of Medicine, University of Southampton, Southampton, UK; 8 Southern Health NHS Foundation Trust, Southampton, UK; 9 Research Department of Clinical, Educational and Health Psychology, University College London, London, UK; 10 Norwich Medical School, University of East Anglia, Norwich, UK; 11 Department of Medicine, Institute of Biomedical Research (IBSAL), University of Salamanca, Salamanca, Spain; 12 Behavioural and Clinical Neuroscience Institute, University of Cambridge, Cambridge, UK; 13 ImmunoPsychiatry, GlaxoSmithKline Research and Development, Brentford, UK; 14 Wellcome Centre for Human Neuroimaging, University College London, London, UK; 15 Medical Research Council Cognition and Brain Sciences Unit, University of Cambridge, Cambridge, UK

**Keywords:** Risk factors, Human behaviour, Epidemiology, Anxiety, Depression

## Abstract

Few studies assessing the effects of COVID-19 on mental health include prospective markers of risk *and* resilience necessary to understand and mitigate the combined impacts of the pandemic, lockdowns, and other societal responses. This population-based study of young adults includes individuals from the Neuroscience in Psychiatry Network (*n* = 2403) recruited from English primary care services and schools in 2012–2013 when aged 14–24. Participants were followed up three times thereafter, most recently during the initial outbreak of the COVID-19 outbreak when they were aged between 19 and 34. Repeated measures of psychological distress (K6) and mental wellbeing (SWEMWBS) were supplemented at the latest assessment by clinical measures of depression (PHQ-9) and anxiety (GAD-7). A total of 1000 participants, 42% of the original cohort, returned to take part in the COVID-19 follow-up; 737 completed all four assessments [mean age (SD), 25.6 (3.2) years; 65.4% female; 79.1% White]. Our findings show that the pandemic led to pronounced deviations from existing mental health-related trajectories compared to expected levels over approximately seven years. About three-in-ten young adults reported clinically significant depression (28.8%) or anxiety (27.6%) under current NHS guidelines; two-in-ten met clinical cut-offs for both. About 9% reported levels of psychological distress likely to be associated with serious functional impairments that substantially interfere with major life activities; an increase by 3% compared to pre-pandemic levels. Deviations from personal trajectories were not necessarily restricted to conventional risk factors; however, individuals with pre-existing health conditions suffered disproportionately during the initial outbreak of the COVID-19 pandemic. Resilience factors known to support mental health, particularly in response to adverse events, were at best mildly protective of individual psychological responses to the pandemic. Our findings underline the importance of monitoring the long-term effects of the ongoing pandemic on young adults’ mental health, an age group at particular risk for the emergence of psychopathologies. Our findings further suggest that maintaining access to mental health care services during future waves, or potential new pandemics, is particularly crucial for those with pre-existing health conditions. Even though resilience factors known to support mental health were only mildly protective during the initial outbreak of the COVID-19 pandemic, it remains to be seen whether these factors facilitate mental health in the long term.

## Introduction

It has been suggested that the ongoing COVID-19 pandemic may be fuelling a mental health crisis, particularly in adolescents and young adults^1,2^. Even though severe disease and any direct neuropsychiatric effects caused by SARS-CoV-2 are uncommon in this age group, the psychological, social, educational, and economic effects of repeated stay-at-home orders, i.e., *lockdowns*, and the emerging concern about the long-term effects of COVID-19 might be expected to exert a toll that could endure long after the pandemic has receded. Despite the need for a rigorous, coordinated response from researchers, few studies on the mental health-related effects of the COVID-19 pandemic within young adults have been longitudinal, or incorporated unbiased measures of pre-pandemic risk and resilience factors. Most have used cross-sectional designs, convenience samples without pre-pandemic comparisons, and have used modified and unvalidated measures^3^. Amongst those which did include population-based data and measures before January 2020, the month the World Health Organisation declared the COVID-19 outbreak a public health emergency of international concern, the overwhelming majority suggests that the initial outbreak of COVID-19 has had a substantial impact on young adults’ mental health, showing a marked increase in symptoms of common mental health disorders such as depression and anxiety compared to pre-pandemic levels^4–7^. A very small number of studies, however, have found no changes in the prevalence of anxiety and depression symptoms^8,9^.

Studies assessing the impact of COVID-19 on mental health have predominately focused on risk factors associated with poorer mental health outcomes, finding that younger age, being female, and having pre-existing health conditions are most commonly related to increased distress and anxiety levels during the pandemic^10–14^. Only a few studies considered data from more than one pre-pandemic assessment, limiting the evidence about how the pandemic has impacted mental health-related trajectories, particularly in adolescence and emerging adulthood^15–17^. Even though some studies assessed coping strategies and potential protective factors^16–18^, we identified no study which tested a range of prospectively-measured resilience factors in this age group.

### What is the added value of this study?

This population-based study addresses several limitations within the current literature by examining both risk *and* resilience in a representative cohort of young adults first assessed at age 14–24 years in 2012–2013 and followed up three times thereafter, most recently during the first lockdown in the United Kingdom in Spring 2020. Surprisingly little research to date has focused on understanding what facilitates mental health and wellbeing during the COVID-19 pandemic. We used latent growth curve modelling of individual trajectories for measurements of psychological distress and mental wellbeing over the first three pre-pandemic assessments (2012–2017) to predict expected mental health at the fourth assessment during the initial outbreak of the COVID-19 pandemic (May 2020). We then quantified pandemic-related effects on mental health as the difference between these expected mental health outcomes and those actually observed (we call these differences ‘extended residuals’ throughout the article).

We hypothesised that observed psychological distress would be higher than expected, and observed mental wellbeing lower than expected, and that this would be more prominent in those at higher risk for common mental disorders such as depression and anxiety. We subsequently assessed the role of prospectively-measured and evidence-based resilience factors at individual, family, and community level. We hypothesised that those who endorsed higher levels across these domains prior to the COVID-19 outbreak, for instance, those reporting high family or friendship support, would deviate less from their expected mental health trajectory compared with those reporting lower levels across these domains.

## Methods

### Study design and participants

Participants were part of the Neuroscience in Psychiatry Network 2400 cohort (NSPN; *n* = 2403) established in 2012 to study the emergence of psychopathology and psychiatric disorders across adolescence and young adulthood. NSPN 2400 is a British cohort that was recruited through primary care services and schools in Greater London, Cambridgeshire, and Peterborough. A total of 2,403 participants were recruited into an age and sex-stratified sample with roughly equal numbers of males and females across five age groups 14–15, 16–17, 18–19, 20–21, and 22–24. The cohort is broadly representative of the youth population across England and Wales when compared to census data of ethnicity, country of birth, parental education, sex, and deprivation. Participants were followed up three times after the baseline, most recently when they were aged between 19 and 34 during the first national lockdown in May 2020.

**Figure 1:**
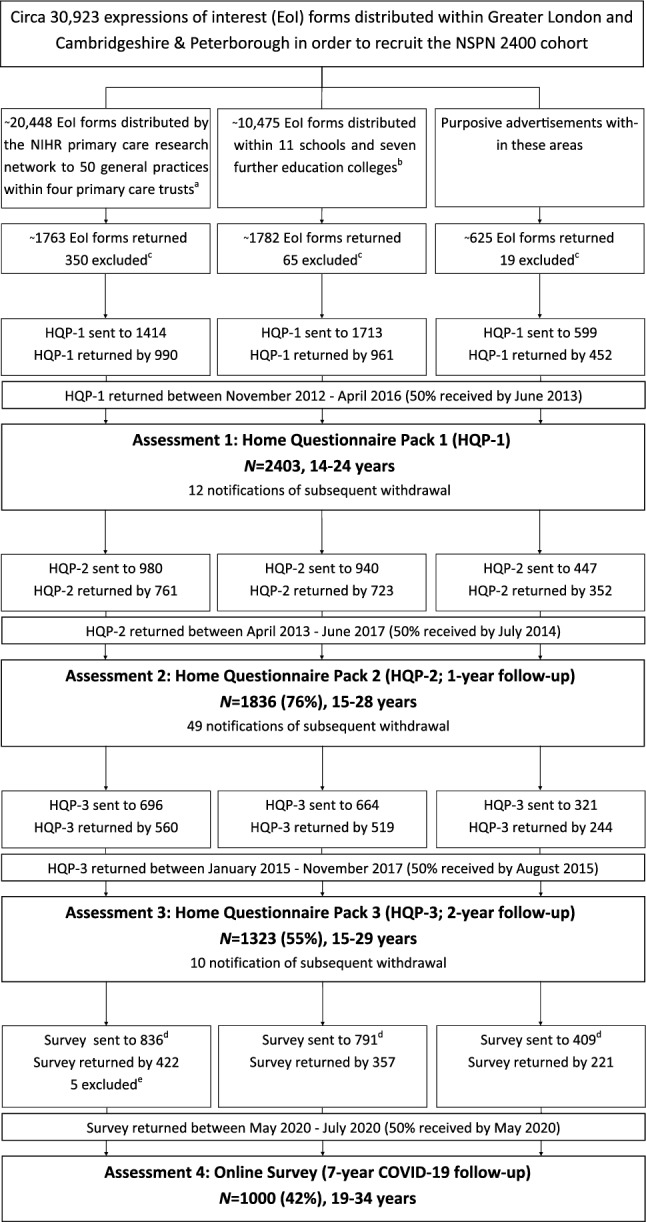
STROBE diagram illustrating the recruitment stages of the NSPN 2400 cohort; adapted from Kiddle et al. (2018). *Eol*  expression of interest, *HQP*  home questionnaire pack; superscript a: 36 practices in Cambridgeshire and Peterborough Primary Care Trust (PCT), 8 in Barnet PCT, 3 in Camden PCT and 3 in Islington PCT; superscript b: schools in Barnet (2), Camden (4), Islington, Tower Hamlets, Haringey, Lambeth and Redbridge (all 1 each), and colleges in Cambridgeshire and Peterborough (6) and Islington (1); superscript c: excluded due to current age beyond scope; superscript d: Assessment 4 was designed as an online survey for which all baseline participants who (a) had a valid email address, and (b) had not withdrawn consent in previous assessments, have been invited (note that for previous assessments only participants who took part in the preceding assessment were invited); superscript e: excluded due to uncertainty of survey responder identity.

This work follows the STROBE guidelines for cohort studies^19^. For further details on recruitment, measures, and representativeness of the cohort, we refer the reader to the published cohort profile by Kiddle and colleagues^20^, an update is currently in preparation. The STROBE diagram, which includes the most recent follow-up, is shown in Fig. 1.

#### Ethical standards

Ethical approval for this study was granted by the Cambridge East Research Ethics Committee under REC 12/EE/0250 for the first three assessments and REC 16/EE/0260 for the fourth assessment. Informed consent was obtained from all participants and/or their legal guardian(s). The authors assert that all procedures contributing to this work comply with the ethical standards of the relevant national and institutional committees on human experimentation and with the Helsinki Declaration of 1975, as revised in 2008.

### Measures

#### Primary outcome measures

We used the Kessler Psychological Distress Scale (K6)^21,22^ and the short version of the Warwick-Edinburgh Mental Wellbeing Scale (SWEMWBS)^23^ to assess changes in psychological distress and wellbeing across time. Both of these self-report measures were designed for use in epidemiological surveys and have shown good ability to monitor population prevalence and trends in psychological distress and wellbeing.

The K6 consists of a 6-item subset of its longer 10-item version assessing non-specific distress relating to depressive and anxious phenomena over the past four weeks. It shows superior total classification accuracy and precision in discrimination between individuals with and without mental illness compared with its longer 10-item version^21^. Its psychometric properties have been extensively validated in adults and increasingly in adolescence and emerging adults, showing that it accurately detects mood and anxiety disorders in this age group^,^^24,25^. However, it has been suggested that due to the high rates of behaviour disorders in adolescence, the K6 should include indicators of such symptoms to adequately screen for serious mental illness^26^. To date, the scale covers symptoms of depression and anxiety such as feeling nervous, hopeless, restless, or worthless. Participants rate each statement on a 5-point Likert scale ranging from 'none of the time' to 'all of the time'. Scores range from 0 to 24. A score of 13 or higher is used to identify individuals with non-specific serious psychological distress, i.e., they have a high likelihood of having a diagnosable mental health condition severe enough to cause major functional limitations which require treatment^21^. It is estimated that serious mental illness affects about 3–4% of the adult population^27^, prevalence in adolescence is estimated to be slightly higher^28^.

The SWEMWBS is a 7-item shortened version of the 14-item WEMWBS measuring aspects of psychological wellbeing over the past two weeks. It is widely used in population studies due to its robust measurement properties, brevity, and well-established population norms in adults and adolescents^29,30^. Items relate to thoughts and feelings of mental wellbeing such as feeling close to other people or feeling relaxed. Participants rate each item on a 5-point Likert scale ranging from ‘none of the time’ to ‘all of the time’. Scores range from 7 to 35 with higher scores indicating better mental wellbeing. Total raw scores are converted to metric scores for parametric statistical procedures using the conversion table published by Stewart-Brown and colleagues^23^.

We assessed the factor structure of both our primary outcome measures. Confirmatory factor analysis within our study sample showed good fit for a one-factor solution and good reliability for either measure. For further details, see Supplementary Materials 1.

#### Secondary outcome measures

We further assessed depression and anxiety using the Patient Health Questionnaire (PHQ-9)^31^ and the Generalised Anxiety Disorder Questionnaire (GAD-7)^32^. Both measures have been added to the most recent NSPN follow-up due to their common use across primary and secondary mental health care services in the UK. The Improving Access to Psychological Therapies (IAPT) services, for instance, routinely use both the PHQ-9 and the GAD-7 to define clinical cases and monitor recovery rates. We were particularly interested to assess IAPT-defined clinical cases during the initial outbreak of the COVID-19 pandemic as a secondary outcome and as an additional validation procedure of our primary outcome approach. Items for both scales are answered on a 4-point Likert scale ranging from ‘not at all’ to ‘nearly every day’ with total scores ranging from 0 to 27 and 0 to 21 for the PHQ-9 and GAD-7 respectively; IAPT-defined clinical cut-offs are ≥ 10 for depression and ≥ 8 for anxiety^33^.

#### Risk factors

Risk factors for psychological distress (higher K6 scores), and poor mental wellbeing (lower SWEMWBS scores), were divided into two major categories covering commonly reported sociodemographic characteristics associated with common mental illnesses such as depression or anxiety as well as specific pandemic-related risk factors from a questionnaire included in the fourth assessment.

Sociodemographic risk factors comprised younger age, female sex, lower educational attainments, non-white ethnicity, and socio-economic deprivation. Qualifications levels were assessed as in the 2011 Census for England and Wales where low educational attainments are defined as no qualification, or any qualifications below Level 4 which is any qualifications below a first degree (such as, e.g., BSc, BA) or equivalent. Ethnicity was obtained at baseline and first follow-up following the Office for National Statistics (ONS) guidelines for the same census. Index of Multiple Deprivation (IMD) was collected at baseline only and was calculated based on the 2015 English Indices of Deprivation. For our analysis, we used deprivation deciles, calculated by ranking all areas from most deprived to least deprived and then dividing them into ten equal groups. The lowest decile refers to the most deprived and the highest to the least deprived areas across England. The pandemic-related questionnaire obtained information on pre-existing health conditions, living situation, self-isolation status, major childcare commitments, and pandemic-related adverse experiences such as job loss, a major cut in household income, hospitalisation, or death due to COVID-19 or another cause. The pandemic-related risk factors were adapted from questionnaire items developed as part of the COVID-19 Social Study (for more information, visit https://www.covidsocialstudy.org/), with a detailed list of items provided in Supplementary Materials 2.

#### Resilience factors

All resilience factors were chosen based upon a pre-registered systematic review^34^ and subsequent investigations by Fritz and colleagues, using exactly the same measures wherever possible^35–37^. We included seven amendable resilience factors at individual, family, and community levels, all assessed through self-report at baseline. Further details on each scale as well as psychometric properties can be found in Supplementary Materials 3.

At individual level:

1. High self-esteem as assessed using the Rosenberg Self-Esteem Scale^38^, a widely-used 10-item scale measuring positive and negative feelings about the self.
2. Low aggression as assessed through four items of the Antisocial Behavioural Checklist^39^, originally derived from DSM-IV clinical criteria for conduct disorder behaviour.
3. Low expressive suppression as measured by the relevant item on the Antisocial Process Screening Device^40^.
4. Low ruminative brooding as assessed by the relevant item in the Short Leyton Obsessional Inventory^41^.

At family and community level:

5. Positive and involved parenting assessed through six items from the Alabama Parenting Questionnaire^42^ measuring retrospectively perceived parenting practices and parental involvement.
6. High family support and high family cohesion measured through the 12-item general functioning subscale of the McMaster Family Assessment Device^43^, a widely used instrument based on the McMaster’s model of general family functioning.
7. High friendship support as measured by six items of the Cambridge Friendship Questionnaire^44^.

### Statistical analysis

**Figure 2:**
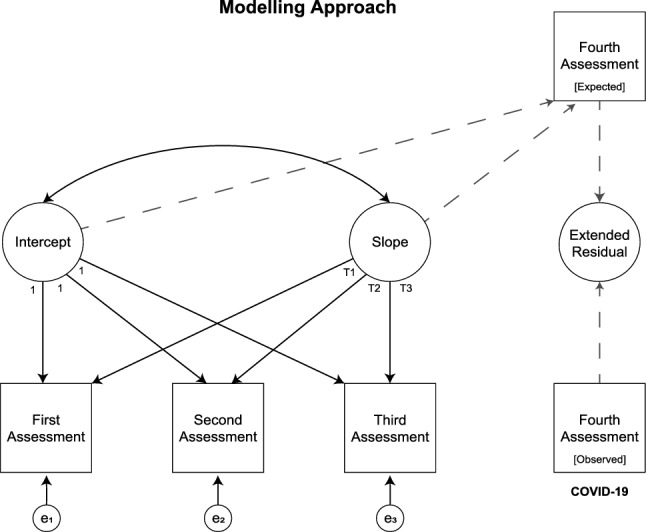
Illustration of latent growth curve modelling and computation of extended residuals; these have been computed for both primary outcome measures separately. Please note we used the exact time point of each assessment as latent slope loadings to account for the length of individual trajectories (cf. Fig. 1 for assessment periods).

We used latent growth curve modelling, a longitudinal analysis technique to estimate growth over time, to fit individual *pre*-pandemic mental health trajectories for total scores of K6 and SWEMWBS. As the NSPN cohort was assessed three times before the initial outbreak of the COVID-19 pandemic, we were limited to estimate linear trends as non-linear estimations require at least four time points. We then used the obtained latent intercept and slope to predict expected total scores at the time point of the fourth assessment. A similar approach has been used previously to estimate the effect of the COVID-19 outbreak on mental health in a community sample of Canadian adolescents^15^. We quantified individual pandemic-related effects by subtracting expected mental health outcomes from those actually observed; we refer to these outcomes as pandemic-related *extended residuals* throughout the article (cf. Fig. 2). The closer an individual scored to zero, the closer their observed mental health score during the pandemic matched their expected mental health score based on their pre-pandemic trajectory through assessments 1–3. An individual with a higher-than-expected K6 score during the pandemic, for instance, would have a positive K6 extended residual score reflecting higher-than-expected psychological distress. Similarly, an individual with a lower-than-expected SWEMWBS score during the pandemic would have a negative SWEMWBS extended residual score reflecting lower-than-expected mental wellbeing. Thus, the pandemic-related extended residuals do not measure cross-sectional mental health at the fourth assessment; rather, they are an *indication* of the psychological response to lockdown, or the initial outbreak of the COVID-19 pandemic in more general. Pandemic-related extended residuals were computed for participants who completed *either* of the primary outcome measures at all four time points. The analytical sample might therefore differ for K6 and SWEMWBS as participants could skip questions or questionnaires if they wished to. These individuals were still included in the cohort to maximise participation and to recognise that they may have valuable data to offer. More details on primary outcome measure availability are provided in Supplementary Materials 1. A comparison of participants who took part in all four assessments with those who dropped out is presented in Supplementary Materials 4.

We assessed mental health-related risk during the pandemic cross-sectionally as well as longitudinally by comparing linear regression models for both observed scores and extended residual scores separately for the K6 and SWEMWBS. Categorical risk factors were binarised as present or absent. We computed both unadjusted as well as adjusted models; the latter adjusted for sociodemographic and pandemic-related risk factors separately. Any *p*-values were corrected for multiple comparisons using the Holm-Bonferroni method^45^. Resilience factors for subsequent analyses were calculated using confirmatory factor analysis, treating item-level data as categorical, analysing polychoric correlation matrices, and using mean- and variance-adjusted weighted least squares as estimator. Further details are provided in Supplementary Materials 3.

The relationship between mental health-related resilience factors and extended residuals was assessed via two regularised partial correlation networks, one for the pandemic-related extended residuals of psychological distress (K6), and another for the extended residuals of mental wellbeing (SWEMWBS). The use of such model approaches has increased substantially over the last decade as they have shown to be a useful tool to explore psychopathology^46^. Results are visualised as network graphs (cf. Fig. 6) where “nodes” (circles or squares) represent variables, in our case, the extended residuals of interest as well as resilience factors, and the “edges” (lines) represent conditional dependencies between two respective variables. Technical details on the model regularisation are provided in Supplementary Materials 6. Network estimation was based on the *full information sample*, i.e., including all possible pairwise associations which includes participants with some missing data. Sensitivity analyses correlating the adjacency matrix, that is the matrix describing the finite graph structure, with its corresponding adjacency matrix for the *complete information sample* (only including participants with no missing data) for both the K6 and SWEMWBS network, showed these matrices were perfectly correlated, supporting the feasibility of this approach (K6: *n* = 632 (full) vs. *n* = 579 (complete); SWEMWBS: *n* = 620 (full) vs. = 566 (complete); *r* = 1.00 for both). We further computed node predictability, here operationalised as R^2^, for the extended residuals to assess the practical relevance of these particular edges. Additional analyses, aimed at evaluating the robustness of the estimated networks are discussed in Supplementary Materials 6 rather than in the article itself.

All data were managed using REDCap^47,48^ electronic data capture tools hosted at the University of Cambridge. REDCap is a secure, web-based application designed to support data capture for research studies, providing (1) an intuitive interface for validated data entry; (2) audit trails for tracking data manipulation and export procedures; (3) automated export procedures for seamless data downloads to common statistical packages; and (4) procedures for importing data from external sources. Analyses were conducted in R [Version 4.0.4]^49^ as well as MPLUS [Version 8.5]^50^. Details on R-packages used are provided in the relevant supplementary materials.

## Results

Of the 2403 members of the NSPN 2400 cohort, 1000 (42%) responded to the fourth assessment during the first national lockdown. Of these, 737 had taken part in all four assessments; their sociodemographic characteristics are described in Table 1. Data on primary outcome measures were not available for all of them as outlined in “Statistical analysis”; the analytical sample for our longitudinal analyses included 632 participants for the K6 and 620 for the SWEMWBS, with an overlap of 598 participants. The corresponding tables of sample characteristics for both sub-samples are presented in Supplementary Materials 4a and 4b. Overall, more male than female participants dropped out, but other key characteristics such as ethnicity, country of birth, parental education, and deprivation remained relatively stable (for further details, see Supplementary Materials 4, Table 6). Baseline mental health as assessed by the primary outcome measures did not differ between the analysed sub-samples when compared with the rest of the cohort.

**Table 1:** Sample characteristics including sociodemographics, pandemic-related factors as well as pre- and mid-pandemic psychological distress and wellbeing for NSPN 2400 cohort participants who took part in all four assessments including the most recent follow-up in May 2020.

	NSPN 2400 cohort sub-sample (*n* = 737)		
	*M (*SD)	*Mdn* (IQR)	*N* (%)
**Sociodemographic factors**			
Age (in years)	25.6 (3.2)	25.0 (23.0–28.0)	–
Sex			
Female	–	–	482 (65.4)
Male	–	–	255 (34.6)
Education^a^			
Below level 4 qualifications (e.g., GCSEs, A-levels)	–	–	192 (26.1)
Level 4+ qualifications (e.g., BA, MA, PhD)	–	–	542 (73.5)
Ethnicity			
White	–	–	583 (79.1)
Non-white	–	–	154 (20.9)
Index of multiple deprivation (IMD; decile)^b^	6.6 (2.7)	7.0 (4.0–9.0)	–
**Pandemic-related factors**			
Pre-existing health conditions			
Yes	–	–	165 (22.3)
No	–	–	569 (77.2)
Living situation			
Alone	–	–	43 (5.8)
With others (e.g., family, friends)	–	–	669 (90.8)
Self-isolation^c^			
Yes	–	–	58 (7.9)
No	–	–	654 (88.7)
Childcare commitments			
Yes	–	–	70 (9.5)
No	–	–	642 (87.1)
Pandemic-related adverse experience			
Yes (e.g., loss of job or income, loss of loved one)	–	–	243 (33.0)
No	–	–	469 (63.6)
**Psychological distress and wellbeing**			
Kessler Psychological Distress Scale			
Pre-pandemic (assessment 1)	6.0 (4.6)	5.0 (3.0–8.0)	–
During pandemic (assessment 4)	6.9 (4.6)	6.0 (3.0–10.0)	–
Short Warwick-Edinburgh Mental Wellbeing Scale			
Pre-pandemic (assessment 1)	22.1 (4.1)	21·5 (19.3—35.0)	–
During pandemic (assessment 4)	21.4 (3.4)	21·5 (19.3—32.6)	–

^a^Qualification levels were assessed as in the census for England & Wales (2011) where Level 4 and above includes at least at least a first degree (or equivalent) and, at most, a doctoral degree such as a PhD.

^b^Index of Multiple Deprivation (IMD) was assessed based on the English Indices of Deprivation (2015) whereas the lowest decile refers to the most deprived 10% of areas in England.

^c^Self-isolation was defined as present for anyone not leaving the house for at least 7 days. Missing data: education (*n* = 3), IMD (*n* = 10), pre-existing health conditions (*n* = 3), living situation (*n* = 25), self-isolation (*n* = 25), childcare commitments (*n* = 25), pandemic-related adverse experience (*n* = 25), Kessler Psychological Distress Scale (K6; *n* = 6 for pre-pandemic, *n* = 13 for during pandemic), Short Warwick-Edinburgh Mental Wellbeing Scale (SWMEBWS; *n* = 10 for pre-pandemic, *n* = 26 for during pandemic).

**Figure 3:**
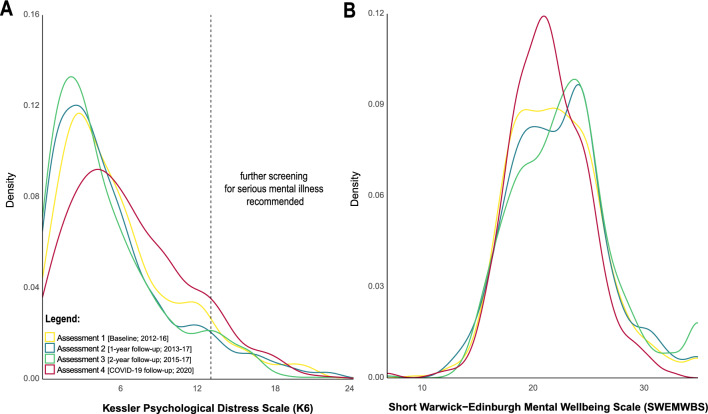
Density distributions for pre-pandemic data collection periods and data collected mid-pandemic for both primary outcome measures; the Kessler Psychological Distress Scale (**A**) and the Short Warwick-Edinburgh Mental Wellbeing Scale (**B**).

We observed an overall shift of score distributions in both primary outcome measures such that the whole population scored *higher* on psychological distress and *lower* on mental wellbeing during the initial outbreak of the COVID-19 pandemic and the first national lockdown when compared with pre-pandemic assessments. This is reflected in Fig. 3 which shows density distributions of both primary outcome measures across all four assessments. The density function of both outcome measures obtained at baseline is significantly different from the density distribution obtained from these measures during the initial outbreak of the COVID-19 pandemic (Kolmogorov–Smirnov test: *D*_K6_ = 0.11, *p* = 0.001, *n* = 632; *D*_SWEMWBS_ = 0.09, *p* = 0.02, *n* = 620), reflecting a shift from lower-to-higher psychological distress and higher-to-lower mental wellbeing during the initial outbreak of the COVID-19 pandemic. This is also the case when comparing the latest pre-pandemic assessment in 2015–17 with the most recent follow-up during the COVID-19 pandemic (Kolmogorov–Smirnov test: *D*_K6_ = 0.22, *p* < 0.001, *n* = 632; *D*_SWEMWBS_ = 0.17, *p* < 0.001, *n* = 620). We further observed that the prevalence of serious mental illness as detected with a K6 scale score ≥ 13 [K6 score range: 0–24] decreased over the first three assessments from 7.4%, 7.0%, to 6.0%, but then increased to 9.0% at the fourth assessment.

Considering the complete sample of the most recent, fourth assessment (*n* = 1000), about three in ten individuals reported depression (*n* = 274 for PHQ-9 ≥ 10) or anxiety (*n* = 263 for GAD-7 ≥ 8) scores that would be considered as clinical cases eligible for treatment under current IAPT guidelines^33^. About two in ten met clinical cut-offs for both (*n* = 190). Data for PHQ-9 and GAD-7 were missing for 48 participants.

**Figure 4:**
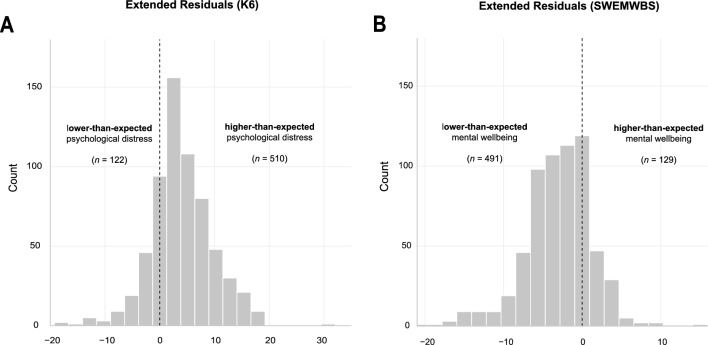
Extended residual scores for both primary outcome measures; the Kessler Psychological Distress Scale (**A**; *n* = 632) and the Short Warwick-Edinburgh Mental Wellbeing Scale (**B**; *n* = 620).

Latent growth curve modelling of individual *pre*-pandemic mental health trajectories showed that psychological distress as measured by the K6 decreased on average by 0.45 points (*y*_K6_ = 5.89—0.45x) at each subsequent assessment; mental wellbeing as measured by the SWEMWBS increased on average by increments of 0.34 (*y*_SWEMWBS_ = 22.14 + 0.34x). This is in line with the trend observed in the density distributions across the first three assessments as displayed in Fig. 3, i.e., overall distress is decreasing, and overall mental wellbeing is increasing over the first three assessments. Fitting individual trends over the first three assessments, subsequently predicting the expected total score at the time point of their fourth assessment and subtracting the expected mental health outcomes from those actually observed (i.e.*,* extended residuals; cf*.* Fig.4), shows that about 8-in-10 individuals score higher-than-expected on the K6 (*n* = 510/632) and lower-than-expected on the SWEMWBS *(n* = 491/620). Please note that this includes individuals close to zero and is therefore solely a descriptive cut-off. The average pandemic-related extended residual score for the K6 was 3.99 (*SD* = 5.67), suggesting that individuals scored about four points higher during the most recent follow-up when compared with expected levels over approximately seven years. Similarly, the average pandemic-related extended residual score for the SWEMWBS was −2.99 (*SD* = 4.25), suggesting that during the first lockdown individuals scored about three SWEMWBS points lower than expected.

### Mental-health related risk

**Figure 5:**
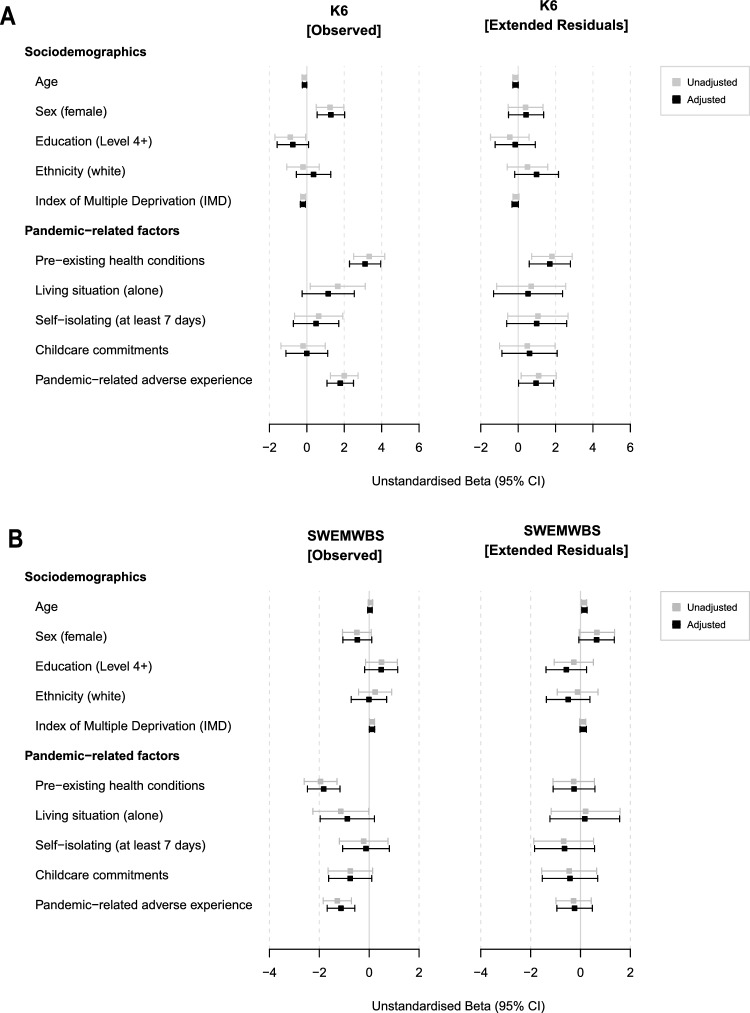
Unstandardised beta coefficients and their 95% confidence intervals for linear models assessing sociodemographic as well as pandemic-related risk factors for both primary outcome measures; the Kessler Psychological Distress Scale (**A**) and the Short Warwick-Edinburgh Mental Wellbeing Scale (**B**). The left panel shows coefficients for the observed scores at Assessment 4 whilst the right panel shows extended residual scores which take into account individual pre-pandemic trajectories.

Linear modelling of risk factors for psychological distress and lower mental wellbeing during the initial outbreak of the COVID-19 pandemic are reported as unstandardised beta coefficient and their 95% confidence intervals (cf. Fig. 5). As unadjusted models did not differ meaningfully from adjusted models (i.e.*,* adjusted for any risk factors within the same category, see Methods 2.3), we report only the latter within the text to ease readability. Detailed results in table format are provided in Supplementary Materials 5. Linear models showed that women scored higher on the K6 compared to men (*b*_[unstd]_ = 1.28, *SE* = 0.37, 95% CI [0.55, 2.02], *p*_adj =_ 0.01). Greater socioeconomic deprivation at baseline (lower IMD decile) was associated with higher self-reported psychological distress, although unstandardised beta coefficients were small (*b*_[unstd]_ = −0.21, *SE* = 0.07, 95% CI [−0.35, −0.08], *p*_adj_ = 0.04). Psychological distress was also significantly higher in individuals who reported any pandemic-related adverse experience (*b*_[unstd]_ = 1.79, *SE* = 0.36, 95% CI [1.08, 2.49], *p*_adj_ < 0.001). Considering individual pandemic-related psychological responses via the extended residuals, however, demonstrated that none of these risk factors were significant predictors.

Pre-existing health conditions (reported during the pandemic) were associated with higher observed K6 scores (*b*_[unstd]_ = 3.11, *SE* = 0.42, 95% CI [2.28, 3.95], *p*_adj_ < 0.001) and higher extended residuals, reflecting higher-than-expected psychological distress (*b*_[unstd]_ = 1.69, *SE* = 0.56, 95% CI [0.59, 2.80], *p*_adj_ = 0.04). Nonetheless, the explanatory power (proportion of the variance explained) in either model was low (R^2^ = 0.12, F(5,617) = 18.26 ,* p* < 0.001 for observed K6 scores and R^2^ = 0.02, F(5,617) = 3.42 ,* p* < 0.01 for corresponding extended residuals). Pre-existing health conditions within this cohort were predominately clinically diagnosed mental health rather than physical conditions. For a detailed breakdown, see Supplementary Materials 4c.

None of the sociodemographic risk factors were a statistically significant predictor of mental wellbeing (SWEMWBS) in linear regression models. Two pandemic-related risk factors significantly predicted observed mental wellbeing scores, i.e., participants with pre-existing health conditions and those who reported pandemic-related adverse experiences, but none of the factors predicted individual pandemic-related psychological responses considering individual pre-pandemic SWEMWBS trajectories (i.e., the extended residuals).

### Mental health-related resilience

**Figure 6:**
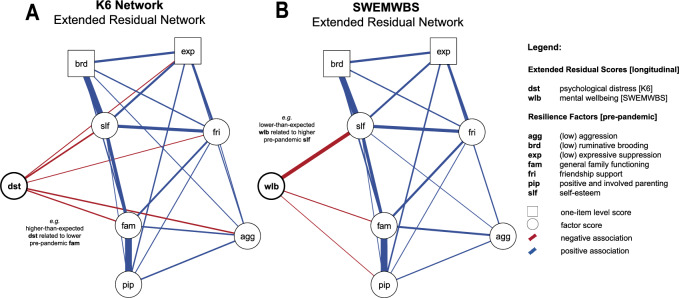
Resilience networks including extended residuals for both primary outcome measures, the Kessler Psychological Distress Scale (**A**; *n* = 632) and the Short Warwick-Edinburgh Mental Wellbeing Scale (**B**; *n* = 620). Extended residuals take into account individual pre-pandemic trajectories, reflecting the deviation from expected mental health whereas greater extended residuals of psychological distress (dst) relate to higher-than-expected distress, and lower extended residuals of mental wellbeing (wlb) relate to lower-than-expected wellbeing**.** Please note that the network layout has been averaged to ease comparison.

The two network graphs for resilience factors in relation to extended residuals of psychological distress and mental wellbeing are presented in Fig. 6. All resilience factors were positively related and coded so that higher scores represent higher resilience. Higher-than-expected psychological distress (dst) during lockdown was related to higher aggression (agg; *r* = −0.07), higher expressive suppression (exp; *r* = −0.03), lower friendship support (fri; *r* = −0.02), lower self-esteem (slf; *r* = −0.08), and lower general family functioning (fam; *r* = −0.06) as assessed at baseline (or all vice versa). The proportion of variance explained by these edges was low (R^2^ = 0.04).

Lower-than-expected mental wellbeing (wlb) during lockdown, on the other hand, was related to higher self-esteem (slf; *r* = −0.25), higher general family functioning (fam; *r* = −0.03), and higher positive and involved parenting (pip; *r* = 0.01) as assessed at baseline (or vice versa). These edges explained about one tenth of the extended residuals’ variance (R^2^ = 0.12). Post-hoc analyses looking at the association between *observed* mental wellbeing mid-pandemic with the above-mentioned resilience factors, however, showed that all were positively correlated (cf. Supplementary Materials 7). Further details assessing the robustness of the estimated networks are provided in Supplementary Materials 6.

## Discussion

This study examined the pandemic-related impact on young adults’ mental health using longitudinal data from a representative cohort of individuals first assessed in 2012–13 at the age of 14–24, followed up three times, most recently at the age of 19–34 during the initial outbreak of the COVID-19 pandemic and the first national lockdown in the United Kingdom. Our results indicate a notable increase in psychological distress and decrease in mental wellbeing compared with expected levels over approximately seven years. During the first COVID-19 wave, about three-in-ten young adults reported depression or anxiety scores classified as clinical cases eligible for psychological talking therapy under current NHS guidelines, about two-in-ten met clinical cut-offs for both. The prevalence of serious mental illness increased from 7.4% at baseline, or 6.0% at the most recent pre-pandemic follow-up completed in 2017, to 9.0% during the initial outbreak of the COVID-19 pandemic. This means that during the initial outbreak of the COVID-19 pandemic nearly 1-in-10 young adults reported levels of psychological distress likely associated with serious functional impairments that substantially interfere with major life activities.

We found that the general increase in psychological distress was ubiquitous and not restricted to those with conventional risk factors. Individuals with pre-existing health conditions, however, were nonetheless disproportionally affected, reporting worse psychological distress, and deviating most strongly from their trajectories compared to their peers. Pre-pandemic self-reported factors which commonly facilitate mental health resilience after adversity were at best mildly protective of individuals' pandemic-related psychological distress response.

The density distributions of both primary outcome measures as well as the pre-pandemic mental health-related trajectories show that psychological distress continuously decreased, and mental wellbeing continuously increased over the first three assessments. These findings are in line with previous longitudinal studies among adolescents and emerging adults, characterising an improvement of general psychological wellbeing during the transition from adolescence to early adulthood^51^. Our data indicate that the initial outbreak of the COVID-19 pandemic led to a notable deterioration from existing mental health-related trajectories, resulting in an overall increase in psychological distress and decrease in mental wellbeing. About 8-in-10 individuals scored higher-than-expected on the K6 and lower-than-expected on the SWEMWBS. Even though this includes some individuals closer to zero where this change is less meaningful, it reflects a clear shift towards worse overall mental health within this sample of young adults. Deviations from mental health trajectories—contrary to expectations—did not differ across age. This could be due to the limited age-range included as previous studies reporting increased risk of mental health problems in young adults often include data across the lifespan^10,52^. The prevalence of individuals with non-specific serious psychological distress increased by 3% compared to the most recent pre-pandemic follow-up. Other measures assessing the prevalence of common mental health disorders such as anxiety or depression potentially requiring treatment, were included only in the most recent follow-up. Hence, we are unable to gain an even more nuanced understanding about the change in psychopathological severity during the pandemic. Nonetheless, the prevalence of both anxiety and depression seems high when compared to existing pre-pandemic epidemiological data within this age group^53–55^.

We observed significantly worse psychological distress during the initial outbreak of the COVID-19 pandemic in female and economically deprived participants. However, these factors did not influence pandemic-driven mental health change from existing trajectories. Similarly, we found that individuals who reported pandemic-related adverse events, such as job loss, or a major cut in household income during the first COVID-19 wave, reported worse psychological distress and mental wellbeing, but much to our surprise, this was not the case when considering pre-pandemic mental health trajectories suggesting that these events may not be independent of pre-existing risk for mental distress and wellbeing. These findings highlight the importance of the availability of pre-pandemic data to properly assess pandemic-driven change in mental health. Results from the UCL COVID-19 Social Study, which started weekly data collection at the end of March 2020, show that even though mental health improved over the course of the pandemic, inequalities were still evident 20 weeks after the start of lockdown^56^. Overall, this suggests that the pandemic has the potential to contribute to a widening of pre-existing mental health-related inequalities.

In line with previous research, individuals with pre-existing health conditions, in this cohort largely driven by an existing diagnosis of depression or anxiety, were at risk of increased psychological distress even after considering individual pre-pandemic trajectories; their vulnerability was magnified^13,52^. Reporting pre-existing health conditions was also related to lower mental wellbeing, however, it did not influence pandemic-driven change from existing trajectories. This supports the importance of assessing both psychological distress and mental wellbeing as they may measure distinct constructs and should not be considered uncritically as being at different ends of a single mental health continuum. Despite the need for future research to address any potential long-term effects of pandemic-related stress within individuals with pre-existing health conditions, our findings highlight the importance of maintaining access to mental health-care services during any new COVID-19 waves, or when preparing for future pandemics.

Overall, none of the resilience factors investigated were protective of pandemic-driven psychological responses, although some factors did show small effects. This suggests that environmental factors that enhance resilience and support mental wellbeing, particularly in response to adverse events, were at best mildly protective of individual psychological responses to the initial outbreak of the COVID-19 pandemic. However, we found a robust and comparatively strong relationship between high pre-pandemic self-esteem and lower-than-expected mental wellbeing during the first COVID-19 lockdown (and vice versa). Nonetheless, individuals with higher pre-pandemic self-esteem displayed higher levels of mental wellbeing compared to their peers with lower pre-pandemic self-esteem. Whilst the way people value and perceive themselves should ideally be independent of others, research has shown that this is often not the case. In fact, the pursuit of self-esteem often relies on external sources, such as approval from others^57^. A possible explanation of our finding may be that the pandemic-induced lack of social contact may have minimised opportunities to reinforce self-esteem at the cost of mental wellbeing. This is further supported by our finding that positive and involved parenting as well as better general family functioning before the pandemic relates, even if only weakly, to lower-than-expected wellbeing during the pandemic. It has previously been hypothesised that that strong social relationships might be a protective factor during the pandemic^58^. This might still be the case; however, our findings show it is not as straight forward and warrants further research. It is possible that other, more direct coping mechanisms, or traits not measured in our study, might support pandemic-related resilience, particularly during the initial outbreak of this pandemic. One study, for instance, has shown that coping strategies such as sticking to a daily routine, regular exercise, and positive reappraisal were associated with less distress and better mental wellbeing in young adults^18^. It remains to be seen whether resilience factors that are known to facilitate mental health after adversity, help to prevent mental health problems in the long-term. In other words, future work will hopefully shed light onto the factors which helped individuals to cope and adapt to this pandemic over the last two years.

### Strengths and limitations

Our study has important strengths, most notably the inclusion of a range of prospectively-measured resilience factors, supporting the approach that such factors should not be studied in isolation^59^. We provide novel insight into markers of risk and resilience necessary to understand and mitigate the combined impacts of the pandemic, lockdowns, and other societal responses. The NSPN cohort is further broadly representative of the youth population across England and Wales when compared to census data of this age group (for further details, we refer the reader to the cohort profile)^20^. Attrition is a limitation, although one shared by other cohorts that include a similar phase of the life course (e.g., ALSPAC)^60^. High attrition rates can lead to bias, as those who drop out of the study may differ from those who remain. For example, the most recent follow-up during the initial COVID-19 outbreak saw 42% of baseline participants return. Although many key characteristics of the cohort, such as ethnicity, country of birth, parental education, and deprivation, remain stable, the disproportionate loss of male participants over time is a concern. However, the homogeneity of the pandemic-related responses across different classifications of participants, such as their sex, for instance, implies that extrapolation of those responses is unlikely to affect the conclusions. The inclusion of pre- and mid-pandemic data is an important strength of this study, however, the availability of ‘only’ three pre-pandemic assessments limits our modelling approach to linear trends within in the framework of latent growth curve models. We explored this limitation by restricting expected scores of primary outcome measures at assessment four to their respective questionnaire boundaries, i.e., if an individual presents with a particular steep slope, we restricted their predicted scores to the minimum or maximum of what the questionnaire allowed (results not reported). Our conclusions remained the same when we used this approach, and we therefore report only the results of the simpler, unrestricted model.

## Conclusions

The initial outbreak of the COVID-19 pandemic has had a significant impact on young adults’ mental health. The effects coincident with lockdown and the early stages of the pandemic were ubiquitous and not necessarily restricted to conventional risk factors. Our findings highlight the importance of the availability of pre-pandemic data to properly assess pandemic-driven change in mental health. The increased risk of poorer mental health outcomes in young adults with pre-existing health conditions, in this cohort largely driven by clinically diagnosed depression or anxiety, further underscores the importance of maintaining access to inclusive mental health-care services during any new COVID-19 waves, or potential future pandemics. In contrast to our predictions, resilience factors known to support mental health, particularly in response to adverse events, had little protective effects on individual psychological responses to the pandemic. It remains to be seen, however, whether such resilience factors facilitate mental health in the long term.

## Supplementary Information

Supplementary Information.

## Data Availability

Data relating to assessments 1–3 analysed during the current study can be requested and downloaded through the portal for the Neuroscience in Psychiatry Network—*NSPN:Open*. Data relating to assessment 4 are not yet publicly available but are available from the corresponding author on reasonable request. NSPN:Open provides access to clinical, cognitive, structural and functional MRI data from a study of healthy adolescent brain development conducted as part of the Neuroscience in Psychiatry Network, a Wellcome Trust-funded collaboration between the University of Cambridge and University College London. These anonymised research data are released to the global scientific research community in accordance with the informed consent of the participants.
