# Author Correction: The impact of the initial COVID-19 outbreak on young adults’ mental health: a longitudinal study of risk and resilience factors

**DOI:** 10.1038/s41598-025-93618-w

**Published:** 2025-03-24

**Authors:** Anna Wiedemann, Jan Stochl, Sharon A. S. Neufeld, Jessica Fritz, Junaid Bhatti, Roxanne W. Hook, NSPN Consortium, Edward Bullmore, Raymond Dolan, Ian Goodyer, Peter Fonagy, Peter Jones, Michael Moutoussis, Tobias Hauser, Sharon Neufeld, Rafael Romero-Garcia, Michelle St. Clair, Petra Vértes, Kirstie Whitaker, Becky Inkster, Gita Prabhu, Cinly Ooi, Umar Toseeb, Barry Widmer, Junaid Bhatti, Laura Villis, Ayesha Alrumaithi, Sarah Birt, Aislinn Bowler, Kalia Cleridou, Hina Dadabhoy, Emma Davies, Ashlyn Firkins, Sian Granville, Elizabeth Harding, Alexandra Hopkins, Daniel Isaacs, Janchai King, Danae Kokorikou, Christina Maurice, Cleo McIntosh, Jessica Memarzia, Harriet Mills, Ciara O’Donnell, Sara Pantaleone, Jenny Scott, Beatrice Kiddle, Ela Polek, Pasco Fearon, John Suckling, Anne-Laura van Harmelen, Rogier Kievit, Sam Chamberlain, Richard A. I. Bethlehem, Ian M. Goodyer, Raymond J. Dolan, Edward T. Bullmore, Samuel R. Chamberlain, Peter Fonagy, Jesus Perez, Peter B. Jones

**Affiliations:** 1 Department of Psychiatry, University of Cambridge, Douglas House, 18B Trumpington Road, Cambridge, CB2 8AH UK; 2 Cambridgeshire and Peterborough NHS Foundation Trust, Cambridge, UK; 3 National Institute for Health Research, Applied Research Collaboration, East of England, Cambridge, UK; 4 Department of Kinanthropology and Humanities, Charles University, Prague, Czechia; 5 Department of Clinical Psychology, Philipps University of Marburg, Marburg, Germany; 6 Max Planck UCL Centre for Computational Psychiatry and Ageing Research, London, UK; 7 Department of Psychiatry, Faculty of Medicine, University of Southampton, Southampton, UK; 8 Southern Health NHS Foundation Trust, Southampton, UK; 9 Research Department of Clinical, Educational and Health Psychology, University College London, London, UK; 10 Norwich Medical School, University of East Anglia, Norwich, UK; 11 Department of Medicine, Institute of Biomedical Research (IBSAL), University of Salamanca, Salamanca, Spain; 12 Behavioural and Clinical Neuroscience Institute, University of Cambridge, Cambridge, UK; 13 ImmunoPsychiatry, GlaxoSmithKline Research and Development, Brentford, UK; 14 Wellcome Centre for Human Neuroimaging, University College London, London, UK; 15 Medical Research Council Cognition and Brain Sciences Unit, University of Cambridge, Cambridge, UK

Correction to: *Scientific Reports* 10.1038/s41598-022-21053-2, published online 05 October 2022.

The original version of this Article contained an omission in the Methods section, under the subheading ‘Risk factors’.

“A detailed list of pandemic-related questionnaire items is provided in Supplementary Materials 2.”

now reads:

“The pandemic-related risk factors were adapted from questionnaire items developed as part of the COVID-19 Social Study (for more information, visit www.covidsocialstudy.org/), with a detailed list of items provided in Supplementary Materials 2.”

In addition, the Funding section was incomplete. The Funding section now reads:

“Data collection was supported by a strategic award from the Wellcome Trust (095844/Z/11/Z) to the University of Cambridge (IMG, ETB, PBJ) and University College London (RJD, PF). Data management was supported by the NIHR Cambridge Bioresource and data analysis by the NIHR ARC East of England. These funders had no role in determining our study design, hypotheses, interpretation, or the writing of this report. AW, JP, and PBJ were supported by the NIHR ARC East of England at Cambridgeshire and Peterborough NHS Foundation Trust. JF was supported by the NIHR Cambridge Biomedical Research Centre; ETB by an NIHR Senior Investigator award. SRC role in this study was funded by a Wellcome Trust Clinical Fellowship (110049/Z/15/Z & 110049/Z/15/A) which also co-supported data collection for the fourth assessment. The NSPN COVID-19 2020 follow-up survey included a collection of items derived from questionnaire material developed as part of the COVID-19 Social Study, which was supported by the Nuffield Foundation (WEL/FR-000022583), the MARCH Mental Health Network funded by the Cross-Disciplinary Mental Health Network Plus initiative supported by UK Research and Innovation (ES/S002588/1), and the Wellcome Trust (221400/Z/20/Z and 205407/Z/16/Z).”

The original Article has been corrected.

