# Supplementary material for: The impact of the initial COVID-19 outbreak on young adults’ mental health: a longitudinal study of risk and resilience factors
Source: Sci Rep. 2022 Oct 5;12:16659. doi: 10.1038/s41598-022-21053-2 (PMC9533974; doi:10.1038/s41598-022-21053-2)
Supplement: Supplementary file 1 — Supplementary Information. [file 41598_2022_21053_MOESM1_ESM.pdf]

Supplementary Materials

The impact of the initial COVID-19 outbreak on young adults' mental health: A longitudinal study of risk and resilience factors

Anna Wiedemann[1,2,3], Jan Stochl[1,2,4], Sharon AS Neufeld[1], Jessica Fritz[1,5], Junaid Bhatti[1], Roxanne W Hook[1], NSPN Consortium*, Ian M Goodyer[1], Raymond J Dolan[6], Edward T Bullmore[1], Samuel R Chamberlain[7,8], Peter Fonagy[9], Jesus Perez[1,2,3,10,11], & Peter B Jones[1,2,3]

[1] Department of Psychiatry, University of Cambridge, UK
[2] Cambridgeshire and Peterborough NHS Foundation Trust, UK
[3] National Institute for Health Research, Applied Research Collaboration, East of England, UK
[4] Department of Kinanthropology and Humanities, Charles University, Czechia
[5] Department of Clinical Psychology, Philipps University of Marburg, Germany
[6] Max Planck UCL Centre for Computational Psychiatry and Ageing Research, UK
[7] Department of Psychiatry, Faculty of Medicine, University of Southampton, UK
[8] Southern Health NHS Foundation Trust, UK
[9] Research Department of Clinical, Educational and Health Psychology, University College London, UK
[10] Norwich Medical School, University of East Anglia, UK
[11] Institute of Biomedical Research (IBSAL), Department of Medicine, University of Salamanca, Spain

*A list of authors and their affiliations can be found at the end of the main article.

Corresponding Author: Anna Wiedemann, Department of Psychiatry, University of Cambridge, Douglas House, 18B Trumpington Road, Cambridge, CB2 8AH, United Kingdom
Email: aw778@medschl.cam.ac.uk

Overview

Supplementary Materials 1 | pp. 02-06

Primary Outcome Measures: This section provides further details on both primary outcome measures including a list of items, data availability across assessments as well as a detailed report of confirmatory factor analysis results.

Supplementary Materials 2 | p. 07

Pandemic-Related Risk Factors: This section provides a list of all pandemic-related items including their possible answer options. Please note that items were binarised for linear regression analyses.

Supplementary Materials 3 | pp. 08-13

Resilience Factors: This section provides a detailed overview of the computation of resilience factors used in the network models. We report confirmatory factor analysis results including fit and reliability indices for each scale, a list of included items as well as further details on the two item-level resilience factors.

Supplementary Materials 4 | pp. 14-17

Sub-Sample Comparison & Characteristics: Here we provide a table comparing key characteristics of participants who took part in all four assessments with participants who did not. We further provide sub-sample descriptives as in Table 1 of the article. The final section provides additional descriptives of pandemic-related risk factors.

Supplementary Materials 5 | pp. 18-21

Linear Regression Models: This section provides the raw scores of the linear regression analyses displayed in figure format within the article. It is divided into two sub-sections, one reporting on the results for the K6 and one for the SWEMWBS models.

Supplementary Materials 6 | pp. 22-34

Regularised Partial Correlation Networks: This section provides a more detailed account of the employed network analyses. It includes two sub-sections reporting association and adjacency matrices and corresponding graphs as well as accuracy and stability checks for both network models separately.

Supplementary Materials 7 | pp. 35-36

Further Correlational Analyses: This final section includes association matrices for both primary outcome measures as observed mid-pandemic as opposed to their extended residuals as in the previous supplements.

References | pp. 37-38

Supplementary Materials 1

Primary Outcome Measures

To verify the underlying factor structure of our primary outcome measures, we used confirmatory factor analysis (CFA) on the NSPN baseline sample (data available: $n_{[\text{K6}]} = 2376$; $n_{[\text{SWEMWBS}]} = 2368$). Analyses were performed using the *R* package *MplusAutomation* [Version 0.8][1] which acts as programming interface to MPLUS, a powerful statistical modelling software primarily known for its latent variable modelling capabilities.[2] We treated item-level data as categorical, analysing the polychoric correlation matrices, and using mean- and variance-adjusted weighted least squares (WLSMV) as estimator. We assessed goodness-of-fit using comparative as well as absolute fit indices.[3–5] More specifically, we used Comparative Fit Index (CFI) as well as Tucker-Lewis Index (TLI) and considered values of $\geq$0.95 as good fit. We further assessed Standardised Root Mean Squared Residual (SRMR) and Root Mean Squared Error of Approximation (RMSEA) with a cut-off of $<$0.08, preferably lower than that. Chi-square test results are also reported in the tables below. We did not, however, rely on chi-square test results to assess model fit due their sensitivity to sample size. We further examined modification indices where appropriate to identify potential improvements in model fit. Reliability was calculated using coefficient omega. Please note that guidelines of model fit indices were developed under normal-theory maximum likelihood with continuous data. To date, there is a lack of investigation into appropriate fit indices for models with categorical data and applying conventional cut-offs can sometimes indicate better model fit than is truly the case.[6] Exercising caution as well as assessing a range of different fit indices when evaluating approximate fit with categorical data is therefore of importance.

Kessler Psychological Distress Scale (K6)

During the last 30 days, about how often did you feel (...)

- Item 1: (...) nervous?
- Item 2: (...) hopeless?
- Item 3: (...) restless or fidgety?
- Item 4: (...) that everything was an effort?
- Item 5: (...) so sad that nothing could cheer you up?
- Item 6: (...) feel worthless?

Short Warwick-Edinburgh Mental Well-being Scale (SWEMWBS)

Over the last two weeks, (...)

- Item 1: (...) I've been feeling optimistic about the future.
- Item 2: (...) I've been feeling useful.
- Item 3: (...) I've been feeling relaxed.
- Item 4: (...) I've been dealing with problems well.
- Item 5: (...) I've been thinking clearly.
- Item 6: (...) I've been feeling close to other people.
- Item 7: (...) I've been able to make up my own mind about things.

Data Availability

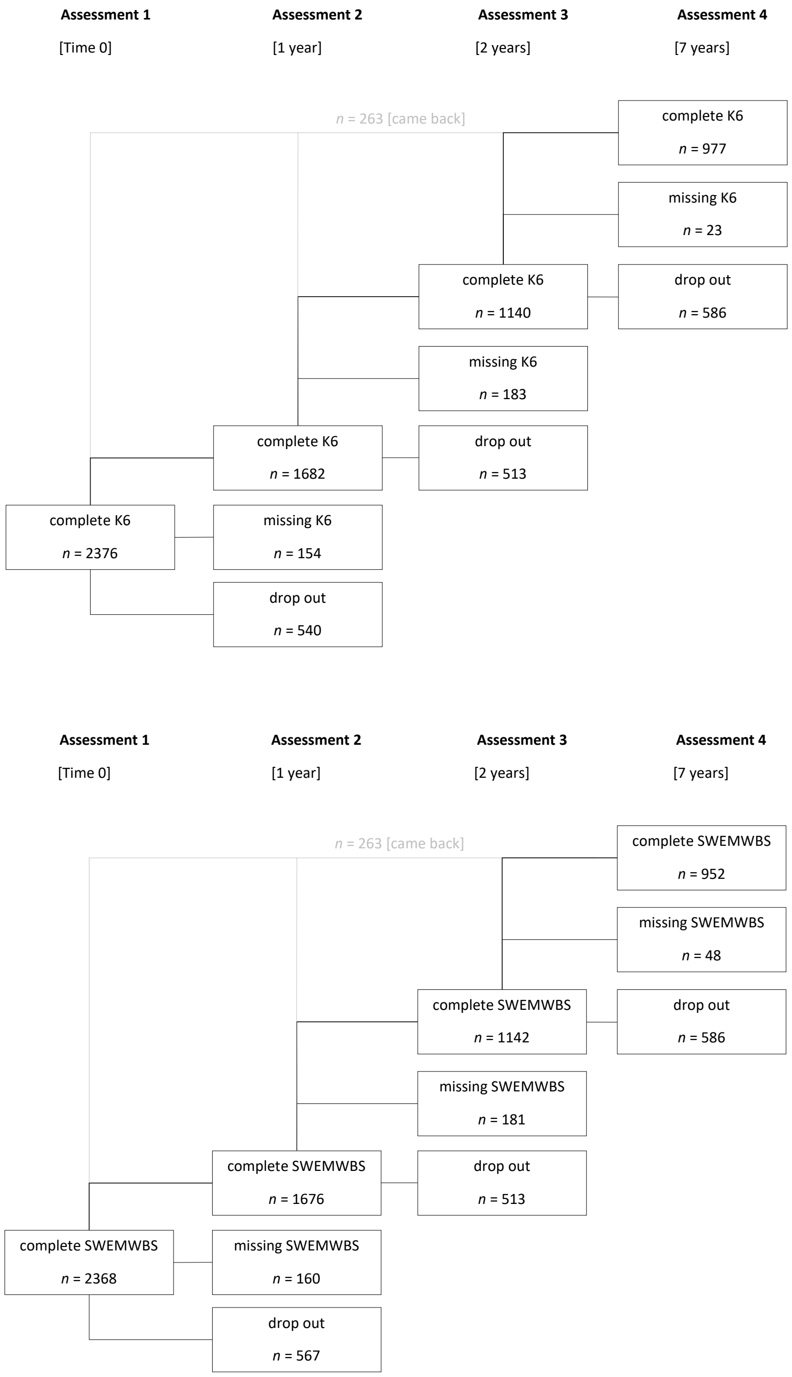

Figure 1: Availability of K6 (top) and SWEMWBS (bottom) data across all assessments. Please note that for the fourth assessment participants from all preceding assessments were invited compared to the third assessment where only participants from the second assessment were sent follow-up invitations.

Correlation Matrix & Factor Loadings (K6; Assessment 1)

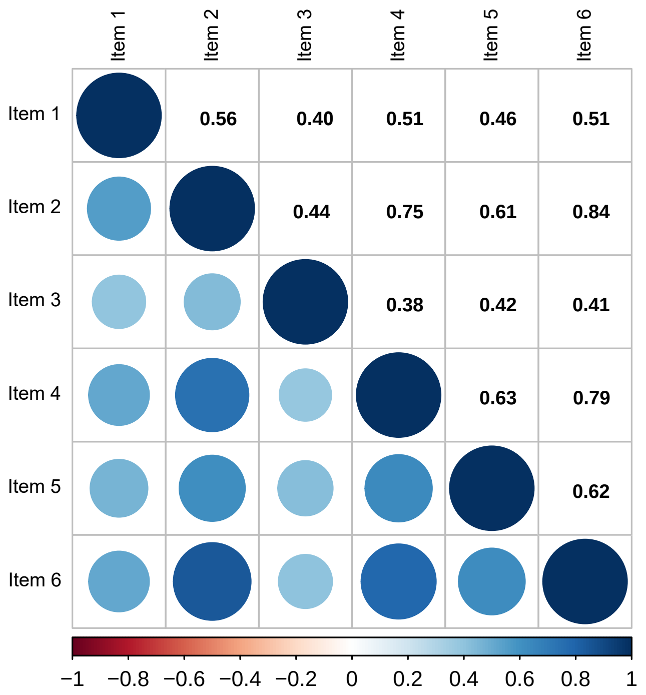

Figure 2: Polychoric correlation matrix of K6 items.

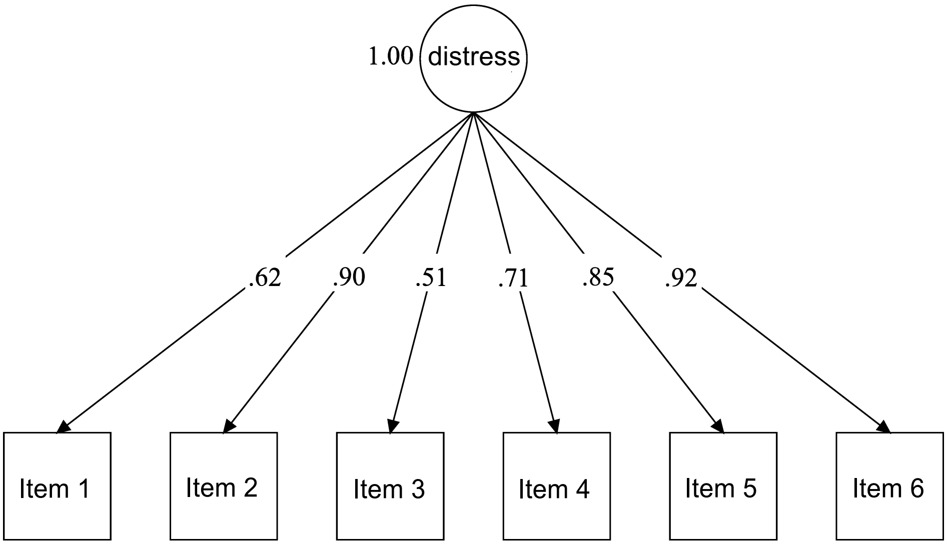

Figure 3: Standardised factor loadings of K6 items.

Correlation Matrix & Factor Loadings (SWEMWBS; Assessment 1)

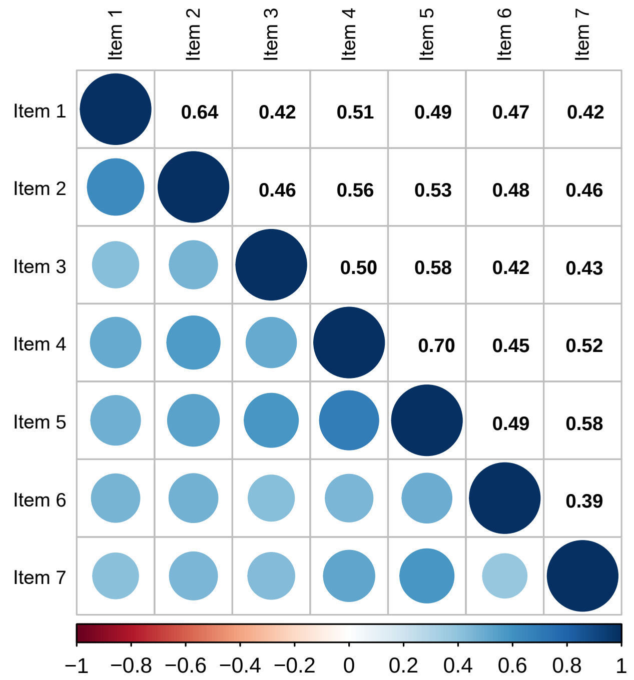

Figure 4: Polychoric correlation matrix of SWEMWBS items.

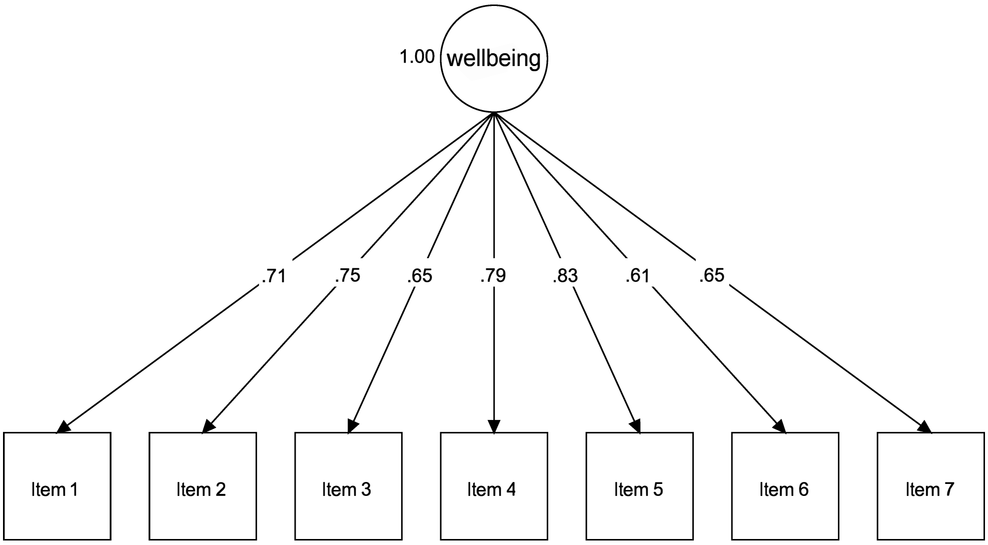

Figure 5: Standardised factor loadings of SWEMWBS items.

Confirmatory Factor Analysis (CFA)

Table 1 shows reasonably good model fit for both the K6 as well as the SWEMWBS. We carefully examined modification indices (MI), adding covariance between the error terms where appropriate, but also limiting such modifications to an absolute minimum due to the small number of items per scale. Modification indices for the one-factor K6 model indicated that model fit would be improved by adding covariance between the error terms for item 1 (feeling nervous) and item 3 (feeling restless; $MI = 52.57$), both referring to similar anxiety-related symptoms. Similarly, adding covariance between the error terms for item 1 (feeling optimistic) and item 2 (feeling useful) within the one-factor SWEMWBS model would significantly improve model fit ($MI = 326.89$). Fit and reliability indices for both unmodified and modified models are displayed in Table 1. Overall, both modified models show reasonable, although not perfect, fit to treat both scales as unidimensional constructs for the purpose of our study.

Table 1: Model fit and reliability indices ($n_{\mathrm{K6}} = 2376$; $n_{\mathrm{SWEMWBS}} = 2368$).

	K6	SWEMWBS
**Unmodified Model**		
χ^2 *(df)*	159.51 (9)	479.30 (14)
CFI	0.99	0.97
TLI	0.99	0.95
SRMR	0.02	0.03
RMSEA [95% CI]	0.08 [0.07,0.10]	0.12 [0.11,0.13]
coefficient ω	0.89	0.88
**Modified Model**		
χ^2 *(df)*	119.40 (8)	163.65 (13)
CFI	0.99	0.99
TLI	0.99	0.98
SRMR	0.02	0.02
RMSEA [95% CI]	0.08 [0.07,0.09]	0.07 [0.06,0.08]
coefficient ω	0.89	0.87

Abbreviations: χ^2 = chi-squared, *df* = degrees of freedom, CFI = Comparative Fit Index, TLI = Tucker-Lewis Index, SRMR = Standardised Root Mean Squared Residual, RMSEA = Root Mean Squared Error of Approximation, 95% CI = 95% Confidence Interval, ω = omega.

Supplementary Materials 2

Pandemic-related Risk Factors

Pre-existing Health Conditions: Do you have any of the following medical conditions? (*Note:* Tick those that apply; references to clinically-diagnosed refers to any diagnosis by a healthcare professional.)

- High blood pressure
- Diabetes
- Heart disease
- Lung disease (*e.g.*, asthma or COPD)
- Cancer
- Another clinically-diagnosed chronic physical health condition
- Clinically-diagnosed depression
- Clinically-diagnosed anxiety
- Another clinically-diagnosed mental health problem
- A disability that affects my ability to leave the house
- Any other disability
- None of the above

Living Situation: During the Covid-19 lockdown, which option best describes your main living situation?

- Living alone
- Living with partner
- Living with partner and other family member(s)
- Living with other family member(s)
- Living with friend(s)
- Living in shared accommodation
- Other

Self-isolation Status: In the past 7 days, how many days have you been self-isolating (not leaving the house)? [answer options: 0-7 days]

Childcare Commitments: During the Covid-19 lockdown have you had major childcare commitments? (*e.g.*, looking after your child/children, or other people's child/children, in your home? [answer options: Yes/No]

Pandemic-related Adverse Experiences: Have you experienced any of the following in the past month?

- Lost your job/been unable to do paid work
- Your spouse/partner lost their job or was unable to do paid work
- Major cut in household income (*e.g.*, due to you or your partner being furloughed/put on leave/not receiving sufficient work)
- Unable to pay bills/rent/mortgage
- Evicted/lost accommodation
- Unable to access sufficient food
- Unable to access required medication
- Somebody close to you is ill in hospital (due to Covid-19 or another illness)
- You lost somebody close to you (due to Covid-19 or another cause)
- None of the above

Supplementary Materials 3

Resilience Factors

Resilience factor scores were estimated based on the full information sample at baseline following a similar methodological approach as discussed by Fritz and colleagues.[7–10] We used confirmatory factor analysis (CFA) to assess factorial validity as well as reliability for each scale. Where appropriate, for instance, where little consensus about a scale's factor structure exists, we compared several models to determine the model of best fit within our sample. We followed the same procedure as discussed in Supplementary Materials 1, *i.e.*, treating item-level data as categorical, analysing polychoric correlation matrices, and using mean- and variance-adjusted weighted least squares (WLSMV) as estimator. If several models were tested, we used maximum likelihood estimation to calculate Akaike's (AIC) and Bayesian Information Criterion (BIC) for model selection. The following sections provide an overview of performed analyses including a list of items used for each resilience factor.

Alabama Parenting Questionnaire (APQ)

The Alabama Parenting Questionnaire was originally developed by Paul Frick in the early 1990s measuring five dimensions of parenting including parental involvement (10 items), positive parenting (6 items), poor supervision (10 items), inconsistent discipline (6 items), and corporal punishment (3 items).[11] The scale deployed within the NSPN 2400 cohort study is a short version of the original 35-item version. It consists of nine items of the short form reported by Elgar and colleagues as well as the entire original corporal punishment scale and three items from the parental involvement scale.[12]

Exploratory as well confirmatory factor analyses commonly show support for a five-factor solution that is largely consistent with the a priori scale structure, however, four-factor solutions where items of the parental involvement and positive parenting load onto the same factor have also been reported.[13] We were particularly interested in the latter two sub-scales as potential resilience factors for our network analysis as both factors have been reported as modifiable resilience factors in Fritz and colleagues' systematic review but have not been included in further investigations.[7] To examine whether items of these two sub-scales should be treated as two distinct resilience factors, or whether they could be combined into one *positive and involved parenting* resilience factor, we tested both a four- (Model A) and five-factor (Model B) solution.

Both AIC and BIC were slightly lower for the five-factor solution. Model fit indices were similar for both the four- and five-factor solution. Total omega was above 0.80 for both models (Model A: $\omega_{\text{total}} = 0.84$; Model B: $\omega_{\text{total}} = 0.81$). In the five-factor model, omega coefficients were 0.20 and 0.14 for the positive parenting and parental involvement sub-scale respectively. More variance, however, was captured in the four-factor model where both sub-scales were combined into one factor and omega increased to 0.49. Given the high correlation of both sub-scales in the five-factor model ($r = 0.87$) as well as the higher variance captured within the four-factor model, we decided to estimate individual factor scores based on the latter, *i.e.*, we included a combined resilience factor which we labelled 'positive and involved parenting' rather than treating

Table 2: Alabama Parenting Questionnaire model fit indices (n = 2306)

	Model A	Model B
AIC	76491.61	76316.53
BIC	76956.82	76804.71
χ^2 *(df)*	1046.69 (84)	904.07 (80)
CFI	0.96	0.97
TLI	0.95	0.96
SRMR	0.05	0.05
RMSEA	0.07	0.07
RMSEA 95% CI	0.07, 0.07	0.06, 0.07

Note: Model A: Four-factor model, Model B: Five-factor model. *Abbreviations*: AIC = Akaike Information Criterion, BIC = Bayesian Information Criterion, χ^2 = chi-squared, *df* = degrees of freedom, CFI = Comparative Fit Index, TLI = Tucker-Lewis Index, SRMR = Standardised Root Mean Squared Residual, RMSEA = Root Mean Squared Error of Approximation, 95% CI = 95% Confidence Interval.

both as two distinct resilience factors. Please note that we also tested a one-factor solution including the six items of interest only. Comparative model fit and reliability were very similar to the four- and five-factor model, however, absolute model fit was rather poor (RMSEA [95% CI] = 0.10 [0.10,0.11]).

Antisocial Behavioural Checklist (ABC)

To assess the degree of aggression, we used four items of the Antisocial Behavioural Checklist.[14] These were the same four items as used by Fritz and colleagues.[8–10] We estimated individual factor scores based on a fitted one-factor model which showed excellent fit and reliability (n = 2380; χ^2 *(df)* = 3.00 (2), CFI = 1.00, TLI = 1.00, SRMR = 0.02, RMSEA [95% CI] = 0.01 [0,0.01], and ω_{total} = 0.88).

Cambridge Friendship Questionnaire (CFQ)

To assess friendship support, we used five items of the Cambridge Friendship Questionnaire.[15] These were the same items as used by Fritz and colleagues.[8–10] We estimated individual factor scores based on a fitted one-factor model with added covariance between the error terms for item 1 ("Are you happy with the number of friends you have got at the moment?") and item 5 ("Overall, how happy are you with your friendships?") due to their similar wording. The model showed very good comparative fit and reliability but rather poor absolute fit (n = 2367; χ^2 *(df)* = 71.94 (2), CFI = 0.99, TLI = 0.99, SRMR = 0.02, RMSEA [95% CI] = 0.09 [0.07,0.10], and ω_{total} = 0.86).

Family Assessment Device (FAD)

The original McMaster Family Assessment Device assesses family functioning on seven different sub-scales including problem solving (5 items), communication (6 items), roles (8 items), affective responsiveness (6 items), affective involvement (7 items), behaviour control (9 items), and general functioning (12 items).[16] The general functioning sub-scale, however, has since been evaluated as a single index measure and has also been deployed as such in the NSPN 2400 cohort study. Fritz and colleagues have previously used this scale to assess family support (5 items) and family cohesion/climate (7

Table 3: General family functioning model fit indices (n = 2325)

	Model A	Model B	Model C
AIC	51546.86	50786.94	50414.06
BIC	51822.93	51068.77	50759.14
χ^2 *(df)*	2724.50 (54)	1359.33 (53)	665.69 (42)
CFI	0.93	0.97	0.98
TLI	0.92	0.97	0.98
SRMR	0.05	0.04	0.02
RMSEA	0.15	0.10	0.08
RMSEA 95% CI	0.14, 0.15	0.10, 0.11	0.08, 0.09

Note: Model A: One-factor model, Model B: Two-factor model (correlated), Model C: Bi-factor model with two specific factors. *Abbreviations*: AIC = Akaike Information Criterion, BIC = Bayesian Information Criterion, χ^2 = chi-squared, *df* = degrees of freedom, CFI = Comparative Fit Index, TLI = Tucker-Lewis Index, SRMR = Standardised Root Mean Squared Residual, RMSEA = Root Mean Squared Error of Approximation, 95% CI = 95% Confidence Interval.

items).[8–10] We examined model fit for a one-factor solution (Model A), a two-factor solution as used by Fritz and colleagues (Model B) as well as a bi-factor model with two specific factors (Model C). AIC and BIC were lowest for the bi-factor model (Model C) which also showed good comparative fit and reasonable absolute fit. McDonald's hierarchical omega was high ($\omega_{\text{hierarchical}} = 0.90$; $\omega_{\text{total}} = 0.95$). We therefore estimated individual estimates for general family functioning based on this model.

Rosenberg Self-Esteem Scale (RSES)

We assessed self-esteem using the Rosenberg Self-Esteem Scale which measures positive (5 items) and negative (5 items) feelings about the self.[17] Even though psychometric properties of the scale have been widely researched, there is little agreement about its factorial validity. Most commonly reported factor structures include one-factor or two-factor solutions. Fritz and colleagues established two one-factor models for positive and negative self-esteem for their network models, however, noted that both factors measure topologically similar concepts.[8–10] Over the last years, bi-factor solutions measuring global self-esteem with two method factors have shown promising results.[18] We therefore tested four different models including a one-factor model (Model A), a two factor model with uncorrelated (Model B) and correlated (Model C) factors as well as a bi-factor model with two specific method factors (Model D).

Both Model C and Model D showed similar AIC and BIC values as well as similar comparative and absolute model fit. Conceptually, however, a global measure of self-esteem seems more appropriate. A general factor explained the majority of the variance within our sample ($\omega_{\text{hierarchical}} = 0.84$; $\omega_{\text{total}} = 0.96$) with specific factors showing rather low reliability. Our results are in line with previous findings comparing more conventional models with bi-factor models. It has since been suggested that the RSES is likely heavily influenced by methods effects due to item wording.[18] We estimated factor scores based on the bi-factor model, using a global self-esteem factor rather than two distinct factors measuring positive and negative aspects of the concept.

Table 4: Rosenberg self-esteem scale model fit indices (n = 2342)

	Model A	Model B	Model C	Model D
AIC	42308.75	42250.87	40850.68	40787.31
BIC	42539.12	42481.24	41086.80	41075.27
χ^2 *(df)*	2333.16 (35)	15049.31 (35)	368.67 (34)	312.06 (25)
CFI	0.95	0.69	0.99	0.99
TLI	0.94	0.61	0.99	0.99
SRMR	0.07	0.30	0.02	0.01
RMSEA	0.17	0.43	0.07	0.07
RMSEA 95% CI	0.16, 0.17	0.42, 0.43	0.06, 0.07	0.06, 0.07

Note: Model A: One-factor model, Model B: Two-factor model (uncorrelated), Model C: Two-factor model (correlated), Model D: Bi-factor model with two specific factors. *Abbreviations*: AIC = Akaike Information Criterion, BIC = Bayesian Information Criterion, χ^2 = chi-squared, *df* = degrees of freedom, CFI = Comparative Fit Index, TLI = Tucker-Lewis Index, SRMR = Standardised Root Mean Squared Residual, RMSEA = Root Mean Squared Error of Approximation, 95% CI = 95% Confidence Interval.

Summary Descriptives

As mentioned in the introduction to this section, we used the full information sample at baseline to estimate resilience factor scores ($n > 2300$). Exact sample sizes used to estimate individual resilience factors have been reported alongside CFA results in the preceding subsections. For the purpose of this study, however, we were particularly interested in individuals who participated in all assessments and completed either of the primary outcome measures at all four time points. Below we present summary statistics of computed resilience factor scores for both longitudinal analysis samples. We further list all items used to compute these scores. Note that all resilience factors have been coded so that higher scores represent higher resilience.

Table 5: Summary statistics including mean (M), standard deviation (SD), median (Mdn), interquartile range (IQR) as well as missing cases (NA) of computed resilience factor scores for those who had key outcomes of psychological distress (K6) or mental well-being (SWEMWBS) at all four assessments. These are presented separately for each longitudinal analysis sample.

	**K6 (n = 632)**			**SWEMWBS (n = 620)**		
	M (SD)	*Mdn* [IQR]	NA	M (SD)	Mdn [IQR]	NA
APQ	0.04 (0.93)	0.06 [-0.58, 0.65]	19	0.04 (0.94)	0.04 [-0.59, 0.66]	20
ABC	-0.06 (0.38)	0.04 [0.04, 0.04]	2	-0.06 (0.39)	0.04 [0.04, 0.04]	2
CFQ	-0.01 (0.78)	0.06 [-0.56, 0.63]	8	-0.01 (0.81)	0.04 [-0.56, 0.71]	8
FAD	0.05 (0.93)	0.03 [-0.56, 0.71]	14	0.05 (0.94)	0.04 [-0.56, 0.71]	15
RSES	0.03 (0.87)	0.08 [-0.59, 0.65]	11	0.01 (0.87)	0.07 [-0.60, 0.63]	11

Note: All resilience factors are coded so that higher scores indicate higher protection. *Abbreviations:* APQ = Alabama Parenting Questionnaire, ABC = Antisocial Behaviour Checklist, CFQ = Cambridge Friendship Questionnaire, FAD = Family Assessment Device, RSES = Rosenberg Self-Esteem Scale.

List of Items

Alabama Parenting Questionnaire⋆ [5-point Likert scale]

⋆ Sub-scales of interest: PI = Parental Involvement, PP = Positive Parenting

- Your parents tell you that you are doing a good job. [PP]
- You play games or do other fun things with your parents. [PI]
- Your parents ask you about your day in school. [PI]
- Your parents help you with your homework. [PI]
- Your parents compliment you when you have done something well. [PP]
- Your parents praise you for behaving well. [PP]

Antisocial Behavioural Checklist⋆ [4-point Likert scale]

⋆ Items of interest

- I deliberately damaged property (*e.g.*, broke windows, wrote graffiti, started fires).
- I deliberately hurt or threatened someone (*e.g.*, bullying or fighting).
- I have carried or used a weapon in a fight (*e.g.*, a knife or a stick).
- I have deliberately hurt or been cruel to an animal (*e.g.*, a pet).

Cambridge Friendship Questionnaire⋆ [4-point Likert scale]

⋆ Items of interest; *Note:* Item 2 is answered on a 6-point Likert scale

- Are you happy with the number of friends you have got at the moment?
- How often do you arrange to see friends other than at school, college, or work?
- Do you feel that your friends understand you?
- Can you confide in your friends?
- Overall, how happy are you with your friendships?

Family Assessment Device⋆ [4-point Likert scale]

⋆ General functioning sub-scale: FS = Family Support, FC = Family Cohesion/Climate; R = Reversed

- Planning family activities is difficult because we misunderstand each other. [FC, R]
- We cannot talk to each other about sadness we feel. [FS, R]
- We feel accepted for what we are. [FC]
- We don't get along well together. [FC, R]
- We can express feelings to each other. [FS]
- In times of crisis we can turn to each other for support. [FS]
- We avoid discussing our fears and concerns. [FS, R]
- We are able to make decisions about how to solve problems. [FC]
- We confide in each other. [FS]
- There are lots of bad feelings in the family. [FC, R]
- Individuals are accepted for what they are. [FC]
- Making decisions is a problem for our family. [FC, R]

Rosenberg Self-Esteem Scale [4-point Likert scale]

R = Reversed

- At times, I thought I was no good at all. [R]
- I was satisfied with myself.
- I felt I had a number of good qualities.
- I was able to do things as well as most people.
- I felt I did not have much to be proud of. [R]
- I certainly felt useless at times. [R]
- I felt that I was as good as anyone else.
- I wished I could have more respect for myself. [R]
- I felt that I was a failure. [R]
- I took a positive attitude towards myself.

Item-Level Resilience Factors

We further included two item-level resilience factors. Expressive suppression was measured by one item ("You hide your feelings or emotions from others"; 3-point Likert scale) from the Antisocial Process Screening Device.[19] The same item has been used by Fritz and colleagues.[8–10] Ruminative brooding was measured by one item from the Leyton Obsessional Inventory.[20] Please note that one further item of this scale has been used by Fritz and colleagues as well as a further five items from a scale not available in the NSPN 2400 cohort. As both items of the Leyton Obsessional Inventory strongly correlated, we chose the slightly more general worded of two available items ("I worried a lot if I did something not exactly the way I liked." [chosen] compared to "I kept thinking about things that I had done because I wasn't sure that they were the right things to do."; 4-point Likert scale). Item-level data for both expressive suppression and ruminative brooding were not standardised but entered as categorical variables in the network models. As previously, reported all resilience factors, including item-level factors, were coded so that higher scores represent higher resilience.

Supplementary Materials 4

Sub-Sample Comparisons

Table 6: Comparison of key characteristics between participants who took part in all four assessments (persistors) and those who did not (observers).

	**Observers** (n = 1666)	**Persistors** (n = 737)
**Ethnicity**		
White	1301 (78.1%)	583 (79.1%)
Asian	156 (9.4%)	72 (9.7%)
Mixed	109 (6.5%)	43 (5.8%)
Black	73 (4.4%)	32 (4.3%)
Other	24 (1.4%)	7 (0.9%)
Missing	3 (0.2%)	-
**Country of Birth**		
UK	1394 (83.7%)	647 (87.8%)
Non-UK	265 (15.9%)	89 (12.1%)
Missing	7 (0.4%)	1 (0.1%)
**Maternal Education**		
No qualification	59 (3.5%)	27 (3.7%)
Vocational	37 (2.2%)	16 (2.2%)
Level 1-3	580 (34.8%)	279 (37.9%)
Level 4	840 (50.4%)	359 (48.7%)
Missing	150 (9.0%)	57 (7.6%)
**Paternal Education**		
No qualification	67 (4.0%)	34 (4.6%)
Vocational	44 (2.6%)	22 (3.0%)
Level 1-3	503 (30.2%)	237 (32.2%)
Level 4	784 (47.1%)	337 (45.7%)
Missing	268 (16.1%)	107 (14.5%)
**IMD Decile** [Mdn, IQR]	7.0 [4.0-9.0]	7.0 [4.0-9.0]
**Sex**		
Female	805 (48.3%)	482 (65.4%)
Male	861 (51.7%)	255 (34.6%)
**Age** [Mdn, IQR]	18.6 [16.6-21.3]	18.8 [16.6-21.8]
**K6** [Mdn, IQR]	5.0 (3.0-9.0)	5.0 (3.0-8.0)
**SWEMWBS** [Mdn, IQR]	21.5 (19.3-24.1)	21.5 (19.3-25.0)

Note: Education as assessed in the 2011 Census of England & Wales where Level l, 2 and 3 have been collapsed into one group and includes at least a GCSE and/or an A-Level at any grade; Level 4 includes any first degree (or equivalent), and, at most, a doctoral degree. [b] Missing data for continuous variables [Observers/Persistors] are 29/10 for Index of Multiple Depreviation (IMD) decile, 21/6 for K6, and 25/10 for SWEMWBS respectively.

Supplementary Materials 4a

Sub-Sample Characteristics (K6)

Table 7: K6 sub-sample (n = 632) characteristics including sociodemographics, pandemic-related factors as well as pre- and mid-pandemic psychological distress and wellbeing.

	**K6 Sample (n = 632)**		
	M (SD)	*Mdn* (IQR)	*N* (%)
**Sociodemographic factors**			
Age (in years)	25.7 (3.2)	25.0 (23.0-28.0)	-
Sex			
Female	-	-	412 (65.2)
Male	-	-	220 (34.8)
Education[a]			
Below Level 4 qualifications	-	-	155 (24.5)
Level 4+ qualifications	-	-	477 (75.5)
Ethnicity			
White	-	-	498 (21.2)
Non-white	-	-	134 (78.8)
IMD decile[b]	6.5 (2.7)	7.0 (4.0-9.0)	-
**Pandemic-related factors**			
Pre-existing health conditions			
Yes	-	-	132 (20.9)
No	-	-	500 (79.1)
Living situation			
Alone	-	-	39 (6.2)
With others (*e.g.,* family, friends)	-	-	584 (92.4)
Self-isolation[c]			
Yes	-	-	52 (8.2)
No	-	-	571 (90.3)
Childcare commitments			
Yes	-	-	63 (10.0)
No	-	-	562 (88.6)
Pandemic-related adverse experience			
Yes (*e.g.,* loss of job or income)	-	-	212 (33.5)
No	-	-	411 (65.0)
**Psychological distress and wellbeing**			
K6			
Pre-pandemic (assessment 1)	5.9 (4.6)	5.0 (3.0-8.0)	-
During pandemic (assessment 4)	6.8 (4.5)	6.0 (3.0-10.0)	-
SWEMWBS			
Pre-pandemic (assessment 1)	22.3 (4.1)	22.4 (19.3-25.0)	-
During pandemic (assessment 4)	21.6 (3.4)	21.5 (19.3-24.1)	-

Note: (a) Qualification levels were assessed as in the Census for England & Wales (2011) where Level 4 and above includes at least a first degree (or equivalent) and, at most, a doctoral degree such as a PhD. (b) Index of Multiple Deprivation (IMD) was assessed based on the English Indices of Deprivation (2015) whereas the lowest decile refers to the most deprived 10% of areas in England. (c) Self-isolation was defined as present for anyone not leaving the house for at least seven days. *Missing data:* IMD (n = 8), living situation, self-isolation, childcare commitments, pandemic-related adverse experience (each n = 9), pre-pandemic SWEMWBS (n = 9), mid-pandemic SWEMWBS (n = 10).

Supplementary Materials 4b

Sub-Sample Characteristics (SWEMWBS)

Table 8: SWEMWBS sub-sample (n = 620) characteristics including sociodemographics, pandemic-related factors as well as pre- and mid-pandemic psychological distress and wellbeing.

	**SWEMWBS Sample (n = 620)**		
	M (SD)	*Mdn* (IQR)	*N* (%)
**Sociodemographic factors**			
Age (in years)	25.8 (3.2)	26.0 (23.0-28.0)	-
Sex			
Female	-	-	411 (66.3)
Male	-	-	209 (33.7)
Education[a]			
Below Level 4 qualifications	-	-	148 (23.9)
Level 4+ qualifications	-	-	472 (76.1)
Ethnicity			
White	-	-	487 (78.5)
Non-white	-	-	133 (21.5)
IMD decile[b]	6.4 (2.8)	7.0 (4.0-9.0)	-
**Pandemic-related factors**			
Pre-existing health conditions			
Yes	-	-	127 (20.5)
No	-	-	493 (79.5)
Living situation			
Alone	-	-	39 (6.3)
With others (*e.g.,* family, friends)	-	-	581 (93.7)
Self-isolation[c]			
Yes	-	-	53 (8.5)
No	-	-	567 (91.5)
Childcare commitments			
Yes	-	-	64 (10.3)
No	-	-	556 (89.7)
Pandemic-related adverse experience			
Yes (*e.g.,* loss of job or income)	-	-	211 (34.0)
No	-	-	409 (66.0)
**Psychological distress and wellbeing**			
K6			
Pre-pandemic (assessment 1)	6.0 (4.5)	5.0 (3.0-8.0)	-
During pandemic (assessment 4)	6.8 (4.5)	6.0 (3.0-10.0)	-
SWEMWBS			
Pre-pandemic (assessment 1)	22.2 (4.1)	22.0 (19.3-25.0)	-
During pandemic (assessment 4)	21.5 (3.5)	22.5 (19.3-24.1)	-

Note: (a) Qualification levels were assessed as in the Census for England & Wales (2011) where Level 4 and above includes at least a first degree (or equivalent) and, at most, a doctoral degree such as a PhD. (b) Index of Multiple Deprivation (IMD) was assessed based on the English Indices of Deprivation (2015) whereas the lowest decile refers to the most deprived 10% of areas in England. (c) Self-isolation was defined as present for anyone not leaving the house for at least seven days. *Missing data:* IMD (n = 7), pre-pandemic K6 (n = 4).

Supplementary Materials 4c

Additional Descriptives

Figure 6 and Figure 7 provide a breakdown of pre-existing health conditions as well as pandemic-related adverse experiences; both risk factors have been binarised for linear regression analyses. Figure 6 shows that the majority of participants reported clinically diagnosed anxiety and/or depression. Of those who reported more than one pre-existing health condition, anxiety and depression were most often reported together. Pandemic-related adverse experiences were driven by a major cut in household income and/or loss of job followed by losing someone close due to COVID-19 or any other cause. The exact wording of items has been provided in Supplementary Materials 2.

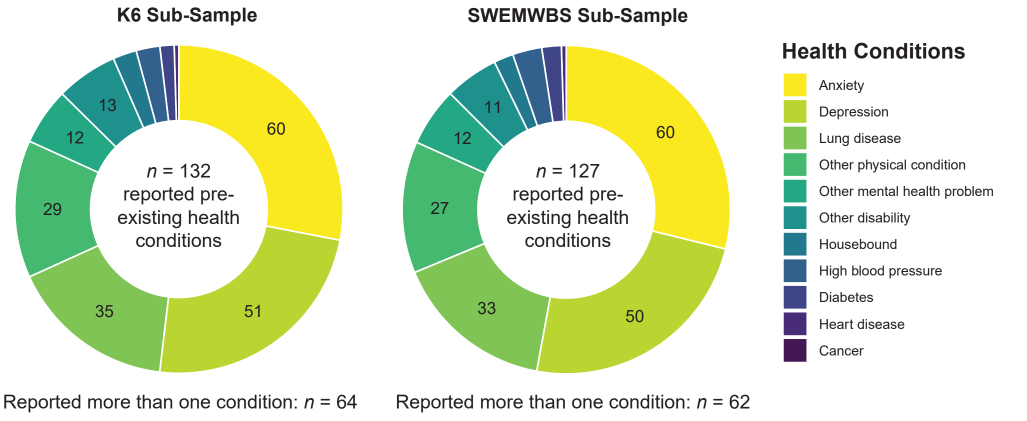

Figure 6: Breakdown of pre-existing health conditions for each of the sub-samples used in the longitudinal analyses. Displayed are only individuals who reported any pre-existing health conditions whereas multiple answers were possible. No pre-existing health condition were reported by the majority of participants ($n_{\text{K6}} = 500$, $n_{\text{SWEMWBS}} = 493$).

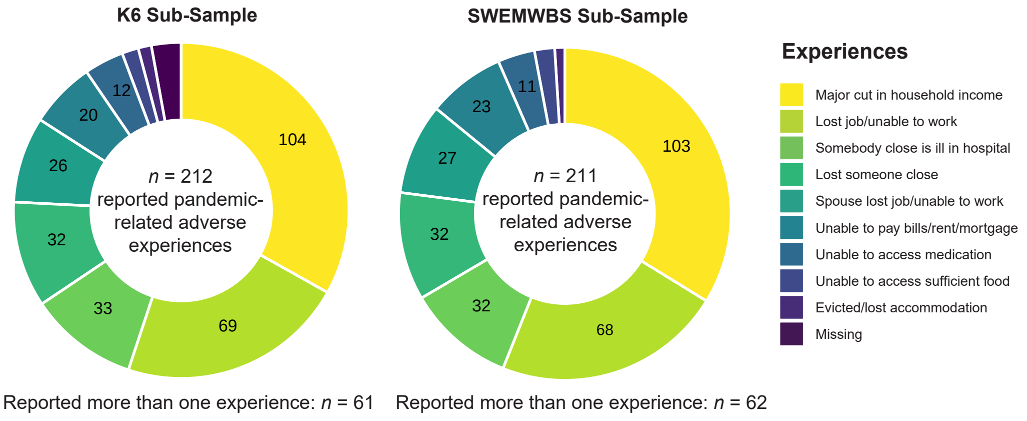

Figure 7: Breakdown of pandemic-related adverse experiences for each of the sub-samples used in the longitudinal analyses. Displayed are only individuals who reported any of these experiences whereas multiple answers were possible. No pandemic-related adverse experiences were reported by the majority of participants ($n_{\text{K6}} = 411$, $n_{\text{SWEMWBS}} = 409$).

Supplementary Materials 5a

Linear Regression Models (K6)

Table 9: Details of linear modelling results for observed psychological distress as measured by Kessler's Psychological Distress Scale and corresponding extended residuals ($n = 632$).

	**K6 [observed, mid-pandemic]**			
	Beta (SE)	[95% CI]	p_{adj}	R^2_{adj}
**Sociodemographic factors**				
**Model A: Age**				<0.01
Intercept	10.46 (1.46)	[7.60, 13.33]	<0.001	
Age	-0.14 (0.06)	[-0.25, -0.03]	0.17	
**Model B: Sex**				0.02
Intercept	5.95 (0.30)	[5.36, 6.55]	<0.001	
Female (vs. male)	**1.24 (0.38)**	[0.50, 1.98]	0.02	
**Model C: Education**[a]				0.01
Intercept	7.43 (0.36)	[6.72, 8.14]	<0.001	
Level 4+ (vs. below Level 4)	-0.89 (0.42)	[-1.71, -0.07]	0.49	
**Model D: Ethnicity**				<0.01
Intercept	6.93 (0.39)	[6.16, 7.69]	<0.001	
White (vs. non-white)	-0.21 (0.44)	[-1.07, 0.66]	1.00	
**Model E: Deprivation**[b]				0.01
Intercept	8.07 (0.46)	[7.16, 8.98]	<0.001	
IMD decile	**-0.20 (0.07)**	[-0.33, -0.07]	0.04	
**Model F: Adjusted**				0.04
Intercept	10.76 (1.51)	[7.78, 13.73]	<0.001	
Age	-0.12 (0.06)	[-0.24, -0.01]	0.49	
Female (vs. male)	**1.28 (0.37)**	[0.55, 2.02]	0.01	
Level 4+ (vs. below Level 4)	-0.75 (0.43)	[-1.59, 0.09]	0.81	
White (vs. non-white)	0.36 (0.47)	[-0.56, 1.28]	1.00	
IMD decile	**-0.21 (0.07)**	[-0.35, -0.08]	0.04	
**Pandemic-related factors**				
**Model A: Pre-existing health**				0.09
Intercept	6.07 (0.19)	[5.69, 6.45]	<0.001	
Pre-existing health conditions	**3.34 (0.42)**	[2.50, 4.17]	<0.001	
**Model B: Living situation**				0.01
Intercept	6.68 (0.19)	[6.31, 7.05]	<0.001	
Living alone (vs. with others)	1.65 (0.75)	[0.18, 3.12]	0.36	
**Model C: Self-isolation**				<0.01
Intercept	6.73 (0.19)	[6.36, 7.10]	<0.001	
Self-isolating (at least 7 days)	0.63 (0.66)	[-0.66, 1.92]	1.00	
**Model D: Childcare**				<0.01
Intercept	6.81 (0.19)	[6.43, 7.18]	<0.001	
Major childcare commitments	-0.20 (0.60)	[-1.39, 0.98]	1.00	
**Model E: Experience**				0.04
Intercept	6.10 (0.21)	[5.67, 6.53]	<0.001	
Pandemic-related adverse experience	**2.00 (0.38)**	[1.26, 2.74]	<0.001	
**Model F: Adjusted**				0.12
Intercept	5.42 (0.24)	[4.95, 5.89]	<0.001	
Pre-existing health conditions	**3.11 (0.42)**	[2.28, 3.95]	<0.001	
Living alone (vs. with others)	1.14 (0.71)	[-0.25, 2.54]	1.00	
Self-isolating (at least 7 days)	0.49 (0.62)	[-0.72, 1.71]	1.00	
Major childcare commitments	0 (0.57)	[-1.12, 1.12]	1.00	
Pandemic-related adverse experience	**1.79 (0.36)**	[1.08, 2.49]	<0.001	

cont.	K6 [extended residuals]			
	Beta (SE)	[95% CI]	p_{adj}	R^2_{adj}
**Sociodemographic factors**				
**Model A: Age**				<0.01
Intercept	7.68 (1.83)	[4.09, 11.28]	<0.001	
Age	-0.14 (0.07)	[-0.28, 0]	0.55	
**Model B: Sex**				<0.01
Intercept	3.73 (0.38)	[2.98, 4.48]	<0.001	
Female (vs. male)	0.40 (0.47)	[-0.53, 1.33]	1.00	
**Model C: Education**[a]				<0.01
Intercept	4.33 (0.46)	[3.43, 5.22]	<0.001	
Level 4+ (vs. below Level 4)	-0.45 (0.52)	[-1.48, 0.58]	1.00	
**Model D: Ethnicity**				<0.01
Intercept	3.60 (0.49)	[2.63, 4.56]	<0.001	
White (vs. non-white)	0.50 (0.55)	[-0.59, 1.58]	1.00	
**Model E: Deprivation**[b]				<0.01
Intercept	4.71 (0.59)	[3.56, 5.86]	<0.001	
IMD decile	-0.11 (0.08)	[-0.28, 0.05]	1.00	
**Model F: Adjusted**				0.01
Intercept	7.68 (1.93)	[3.88, 11.47]	<0.01	
Age	-0.14 (0.07)	[-0.29, 0]	0.69	
Female (vs. male)	0.42 (0.48)	[-0.51, 1.36]	1.00	
Level 4+ (vs. below Level 4)	-0.16 (0.55)	[-1.23, 0.92]	1.00	
White (vs. non-white)	0.99 (0.60)	[-0.19, 2.16]	0.89	
IMD decile	-0.16 (0.09)	[-0.33, 0.01]	0.79	
**Pandemic-related factors**				
**Model A: Pre-existing health**				0.02
Intercept	3.61 (0.25)	[3.12, 4.11]	<0.001	
Pre-existing health conditions	**1.81 (0.55)**	[0.72, 2.89]	0.02	
**Model B: Living situation**				<0.01
Intercept	3.95 (0.24)	[3.48, 4.41]	<0.001	
Living alone (vs. with others)	0.70 (0.94)	[-1.15, 2.54]	1.00	
**Model C: Self-isolation**				<0.01
Intercept	3.90 (0.24)	[3.43, 4.37]	<0.001	
Self-isolating (at least 7 days)	1.06 (0.82)	[-0.56, 2.67]	1.00	
**Model D: Childcare**				<0.01
Intercept	3.94 (0.24)	[3.47, 4.41]	<0.001	
Major childcare commitments	0.49 (0.75)	[-0.99, 1.97]	1.00	
**Model E: Experience**				0.01
Intercept	3.62 (0.28)	[3.07, 4.16]	<0.001	
Pandemic-related adverse experience	1.10 (0.48)	[0.16, 2.04]	0.31	
**Model F: Adjusted**				0.02
Intercept	3.13 (0.32)	[2.51, 3.76]	<0.001	
Pre-existing health conditions	**1.69 (0.56)**	[0.59, 2.80]	0.04	
Living alone (vs. with others)	0.53 (0.94)	[-1.32, 2.37]	1.00	
Self-isolating (at least 7 days)	0.99 (0.82)	[-0.61, 2.59]	1.00	
Major childcare commitments	0.61 (0.75)	[-0.87, 2.08]	1.00	
Pandemic-related adverse experience	0.96 (0.48)	[0.02, 1.90]	0.54	

Note: Table shows unstandardised beta coefficients and standard errors as well as 95% confidence intervals; also shown are adjusted p-values (adjusted for sociodemographic and pandemic-related risk factors separately; for details on the adjustment method used, *see* Holm, 1979) and adjusted R squared.[21] Any significant risk factors are highlighted in bold. *Further notes:* [a] Qualification levels were assessed as in the Census for England & Wales (2011) where Level 4 and above includes at least a first degree (or equivalent) and, at most, a doctoral degree such as a PhD. [b] Index of Multiple Deprivation (IMD) was assessed based on the English Indices of Deprivation (2015) whereas the lowest decile refers to the most deprived 10% of areas in England.

Supplementary Materials 5b

Linear Regression Models (SWEMWBS)

Table 10: Details of linear modelling results for observed mental wellbeing as measured by the Short Warwick-Edinburgh Mental Wellbeing Scale and corresponding extended residuals (n = 620).

	**SWEMWBS [observed, mid-pandemic]**			
	Beta (SE)	[95% CI]	p_{adj}	R^2_{adj}
**Sociodemographic factors**				
**Model A: Age**				<0.01
Intercept	20.21 (1.14)	[17.98, 22.44]	<0.001	
Age	0.05 (0.04)	[-0.04, 0.14]	1.00	
**Model B: Sex**				<0.01
Intercept	21.84 (0.24)	[21.37, 22.31]	<0.001	
Female (vs. male)	-0.49 (0.29)	[-1.07, 0.08]	1.00	
**Model C: Education**[a]				<0.01
Intercept	21.14 (0.28)	[20.58, 21.70]	<0.001	
Level 4+ (vs. below Level 4)	0.49 (0.33)	[-0.14, 1.13]	1.00	
**Model D: Ethnicity**				<0.01
Intercept	21.33 (0.30)	[20.74, 21.92]	<0.001	
White (vs. non-white)	0.24 (0.34)	[-0.43, 0.90]	1.00	
**Model E: Deprivation**[b]				0.01
Intercept	20.78 (0.36)	[20.08, 21.48]	<0.001	
IMD decile	0.11 (0.05)	[0.01, 0.21]	0.45	
**Model F: Adjusted**				0.01
Intercept	19.98 (1.19)	[17.64, 22.32]	<0.001	
Age	0.03 (0.05)	[-0.06, 0.12]	1.00	
Female (vs. male)	-0.48 (0.29)	[-1.06, 0.10]	1.00	
Level 4+ (vs. below Level 4)	0.48 (0.34)	[-0.18, 1.14]	1.00	
White (vs. non-white)	-0.01 (0.36)	[-0.72, 0.70]	1.00	
IMD decile	0.11 (0.05)	[0.01, 0.22]	0.65	
**Pandemic-related factors**				
**Model A: Pre-existing health**				0.05
Intercept	21.91 (0.15)	[21.62, 22.2]	<0.001	
Pre-existing health conditions	**-1.95 (0.33)**	[-2.61, -1.29]	<0.001	
**Model B: Living situation**				<0.01
Intercept	21.59 (0.14)	[21.31, 21.87]	<0.001	
Living alone (vs. with others)	-1.14 (0.57)	[-2.26, -0.02]	0.74	
**Model C: Self-isolation**				<0.01
Intercept	21.53 (0.15)	[21.25, 21.82]	<0.001	
Self-isolating (at least 7 days)	-0.22 (0.50)	[-1.10, 0.75]	1.00	
**Model D: Childcare**				<0.01
Intercept	21.59 (0.15)	[21.31, 21.88]	<0.001	
Major childcare commitments	-0.76 (0.46)	[-1.65, 0.14]	1.00	
**Model E: Experience**				0.03
Intercept	21.95 (0.17)	[21.62, 22.28]	<0.001	
Pandemic-related adverse experience	**-1.28 (0.29)**	[-1.85,-0.71]	<0.001	
**Model F: Adjusted**				0.07
Intercept	22.42 (0.19)	[22.05, 22.78]	<0.001	
Pre-existing health conditions	**-1.82 (0.33)**	[-2.48, -1.17]	<0.001	
Living alone (vs. with others)	-0.88 (0.55)	[-1.97, 0.21]	1.00	
Self-isolating (at least 7 days)	-0.13 (0.48)	[-1.07, 0.81]	1.00	
Major childcare commitments	-0.76 (0.44)	[-1.63, 0.10]	0.94	
Pandemic-related adverse experience	**-1.13 (0.28)**	[-1.69, -0.58]	<0.01	

cont.	SWEMWBS [extended residuals]			
	Beta (SE)	[95% CI]	p_{adj}	R^2_{adj}
**Sociodemographic factors**				
**Model A: Age**				0.01
Intercept	-6.70 (1.39)	[-9.43, -3.97]	<0.001	
Age	0.14 (0.05)	[0.04, 0.25]	0.14	
**Model B: Sex**				<0.01
Intercept	-3.42 (0.29)	[-4.00, -2.85]	<0.001	
Female (vs. male)	0.65 (0.36)	[-0.05, 1.37]	1.00	
**Model C: Education**[a]	-2.78 (0.35)	[-3.47, -2.10]	<0.001	<0.01
Intercept				
Level 4+ (vs. below Level 4)	-0.27 (0.40)	[-1.05, 0.52]	1.00	
**Model D: Ethnicity**				<0.01
Intercept	-2.90 (0.37)	[-3.62, -2.17]	<0.001	
White (vs. non-white)	-0.12 (0.42)	[-0.93, 0.70]	1.00	
**Model E: Deprivation**[b]				<0.01
Intercept	-3.56 (0.44)	[-4.43, -2.70]	<0.001	
IMD decile	0.09 (0.06)	[-0.04, 0.21]	1.00	
**Model F: Adjusted**				0.02
Intercept	-7.35 (1.46)	[-10.22, -4.48]	<0.001	
Age	0.16 (0.06)	[0.05, 0.27]	0.10	
Female (vs. male)	0.65 (0.36)	[-0.06, 1.36]	1.00	
Level 4+ (vs. below Level 4)	-0.57 (0.41)	[-1.38, 0.24]	1.00	
White (vs. non-white)	-0.49 (0.44)	[-1.37, 0.38]	1.00	
IMD decile	0.11 (0.07)	[-0.02, 0.24]	1.00	
**Pandemic-related factors**				
**Model A: Pre-existing health**				<0.01
Intercept	-2.93 (0.19)	[-3.31, -2.55]	<0.001	
Pre-existing health conditions	-0.27 (0.42)	[-1.10, 0.56]	1.00	
**Model B: Living situation**				<0.01
Intercept	-3.00 (0.18)	[-3.34, -2.65]	<0.001	
Living alone (vs. with others)	0.21 (0.70)	[-1.17, 1.59]	1.00	
**Model C: Self-isolation**				<0.01
Intercept	-2.93 (0.18)	[-3.28, 2.58]	<0.001	
Self-isolating (at least 7 days)	-0.67 (0.61)	[-1.87, 0.52]	1.00	
**Model D: Childcare**				<0.01
Intercept	-2.94 (0.18)	[-3.29, -2.59]	<0.001	
Major childcare commitments	-0.46 (0.56)	[-1.56, 0.665]	1.00	
**Model E: Experience**				<0.01
Intercept	-2.89 (0.21)	[-3.30, -2.48]	<0.001	
Pandemic-related adverse experience	-0.28 (0.36)	[-0.98, 0.43]	1.00	
**Model F: Adjusted**				<0.01
Intercept	-2.77 (0.24)	[-3.23, -2.30]	<0.001	
Pre-existing health conditions	-0.26 (0.43)	[-1.10, 0.58]	1.00	
Living alone (vs. with others)	0.17 (0.71)	[-1.22, 1.57]	1.00	
Self-isolating (at least 7 days)	-0.64 (0.61)	[-1.84, 0.56]	1.00	
Major childcare commitments	-0.42 (0.57)	[-1.53, 0.69]	1.00	
Pandemic-related adverse experience	-0.24 (0.36)	[-0.95, 0.48]	1.00	

Note: Table shows unstandardised beta coefficients and standard errors as well as 95% confidence intervals; also shown are adjusted p-values (adjusted for sociodemographic and pandemic-related risk factors separately; for details on the adjustment method used, *see* Holm, 1979) and adjusted R squared.[21] Any significant risk factors are highlighted in bold. *Further notes:* [a] Qualification levels were assessed as in the Census for England & Wales (2011) where Level 4 and above includes at least a first degree (or equivalent) and, at most, a doctoral degree such as a PhD. [b] Index of Multiple Deprivation (IMD) was assessed based on the English Indices of Deprivation (2015) whereas the lowest decile refers to the most deprived 10% of areas in England.

Supplementary Materials 6

Regularised Partial Correlation Networks

We estimated network models separately for the pandemic-related distress (K6) and mental wellbeing (SWEMWBS) response as measured by the respective extended residuals. Both models are visualised as network graphs in the article (Figure 5) whereas *nodes* (circles or squares) represent variables, in our case, the extended residual of interest and resilience factors, and *edges* (lines) represent conditional dependencies, estimated as partial correlations, between two respective variables.

A key element of network estimation is not only the identification of conditional dependencies, but also the identification of edges that are truly zero. A fully connected network, for instance, is hard to interpret and not very helpful in gaining insight into predictive and potentially causal relationships. To estimate a parsimonious and interpretable network, we used the R-package *qgraph* [version 1.6.9].[22] We estimated both networks via the Extended Bayesian Information Criterion (EBIC) graphical *least absolute shrinkage and selection operator* (LASSO) using polychoric correlations for item-level resilience factors (*i.e.,* low expressive suppression/low ruminative brooding, see Supplementary Materials 3).[23] The graphical LASSO sets edges that are likely to be spurious to exactly zero, resulting in a sparse network.[24] It estimates a collection of networks where the optimal network is chosen by minimising the model selection criterion, *i.e.,* EBIC. The latter requires using a hyperparameter gamma (γ) which controls the degree to which simpler models are preferred; it is usually set between 0-0.5 where higher values indicate simpler models. We used default settings of $\gamma = 0.5$, ensuring a more conservative approach.

Focusing on the network structure as a whole within the article, we here present additional analyses of centrality indices including node *betweenness*, *closeness*, and *strength.* Such centrality indices can provide information of relative importance of nodes within a specific network structure. Betweenness, for instance, measures how often a specific node acts as bridge along the shortest path between two other nodes. Closeness, on the other hand, acts as an indicator of how close a node is to other nodes within the network. Node strength is measured by the sum of edge weights connected to the node. The use of centrality indices, however, is increasingly de-emphasised as its underlying assumptions, originating from social network theory, may not necessarily correspond to relationships between psychological variables; therefore considerable care is needed in the interpretation of results.[25] We further used non-parametric bootstrapping (N boot = 2000) to assess the robustness of the estimated networks parameters and descriptive statistics. Accuracy and stability checks were conducted using the R-package *bootnet* [version 1.4.3].[26] For further details, we would like to refer the reader to Sacha Eshkamp and Eiko Fried's tutorial on regularised partial correlation network.[27]

In the following pages, we report:

- (...) association and lasso regularised networks and corresponding matrices,
- (...) accuracy and differences of edge-weights,
- (...) centrality indices and their stability.

Supplementary Materials 6a

Further Network Analysis Results (K6)

Association Network

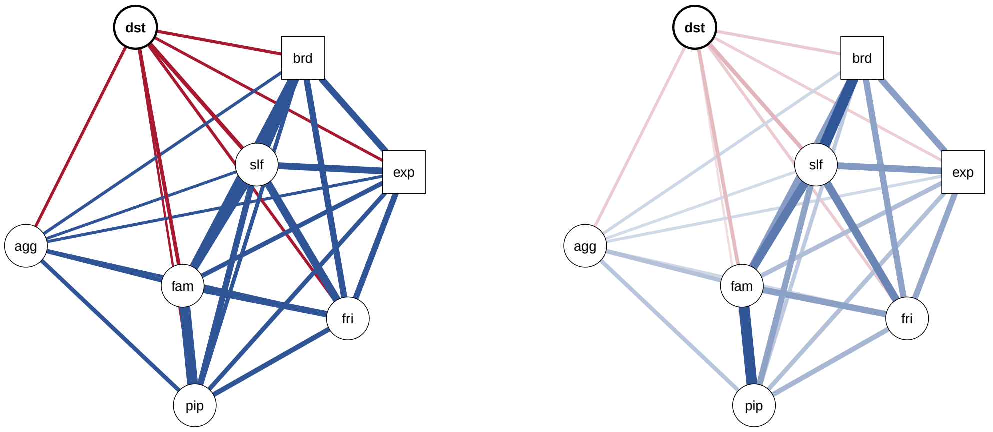

(a) Unfaded Association Network **(b)** Faded Association Network

Figure 8: Association network including pre-pandemic resilience factors and pandemic-related distress response measured by K6 extended residual scores where higher scores indicate higher-than-expected psychological distress during the first national lockdown compared with expected levels over seven years ($n = 632$). All resilience factors are coded so that higher scores indicate higher protection. **Legend**: pandemic-related distress response/K6 extended residuals [**dst**], self-esteem [**slf**], friendship support [**fri**], general family functioning [**fam**], positive and involved parenting [**pip**], low aggression [**agg**], low expressive suppression [**exp**], low ruminative brooding [**brd**]. Nodes surrounded by a circle denote factor scores, nodes surrounded by a square denote single item-level (categorical) scores. Edge colour refers to either positive (blue) or negative (red) relations. Note that within the faded network the colour fades the weaker the edge weight is.

Table 11: Corresonding correlation matrix to Figure 8.

	dst	slf	fri	fam	pip	agg	exp	brd
dst	1.00							
slf	-0.20	1.00						
fri	-0.14	0.44	1.00					
fam	-0.18	0.48	0.34	1.00				
pip	-0.09	0.33	0.26	0.61	1.00			
agg	-0.14	0.13	0.14	0.22	0.21	1.00		
exp	-0.13	0.37	0.32	0.24	0.23	0.14	1.00	
brd	-0.14	0.61	0.34	0.35	0.20	0.15	0.35	1.00

Note: We used the *cor_auto* function of the R-package *qgraph* to automatically compute the appropriate correlation matrix based on polychoric and Pearson correlations. For a legend, see Figure 8.

Lasso Regularised Network

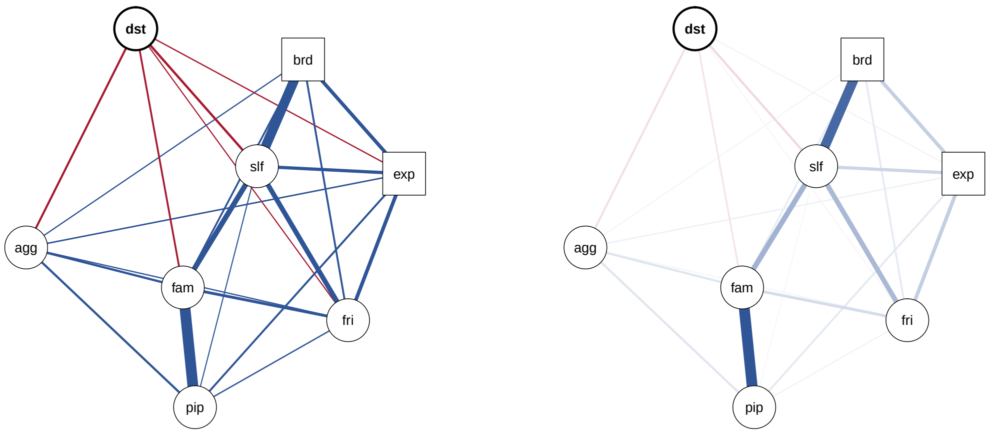

(a) Unfaded Lasso Regularised Network **(b)** Faded Lasso Regularised Network

Figure 9: Lasso regularised network including pre-pandemic resilience factors and pandemic-related distress response measured by K6 extended residual scores where higher scores indicate higher-than-expected psychological distress during the first national lockdown compared with expected levels over seven years ($n = 632$). All resilience factors are coded so that higher scores indicate higher protection. **Legend**: pandemic-related distress response/K6 extended residuals [**dst**], self-esteem [**slf**], friendship support [**fri**], general family functioning [**fam**], positive and involved parenting [**pip**], low aggression [**agg**], low expressive suppression [**exp**], low ruminative brooding [**brd**]. Nodes surrounded by a circle denote factor scores, nodes surrounded by a square denote single item-level (categorical) scores. Edge colour refers to either positive (blue) or negative (red) relations. Note that within the faded network the colour fades the weaker the edge weight is.

Table 12: Corresponding weights matrix for Figure 9.

	dst	slf	fri	fam	pip	agg	exp	brd
dst	0.00							
slf	-0.08	0.00						
fri	-0.02	0.20	0.00					
fam	-0.06	0.22	0.10	0.00				
pip	0.00	0.02	0.03	0.50	0.00			
agg	-0.07	0.00	0.03	0.07	0.07	0.00		
exp	-0.03	0.12	0.14	0.00	0.06	0.04	0.00	
brd	0.00	0.44	0.06	0.05	0.00	0.03	0.14	0.00

Note: For a legend, see Figure 9.

Summary No. 1:

The association network in Figure 8 shows that all resilience factors are positively (blue edges), and mostly strongly (edge thickness), correlated. The negative correlations (red edges) between resilience factors and pandemic-related distress response as measured by the K6 extended residuals mean that higher-than-expected psychological distress was related to lower resilience scores and vice versa. The lasso regularised network model is discussed in detail within the main article.

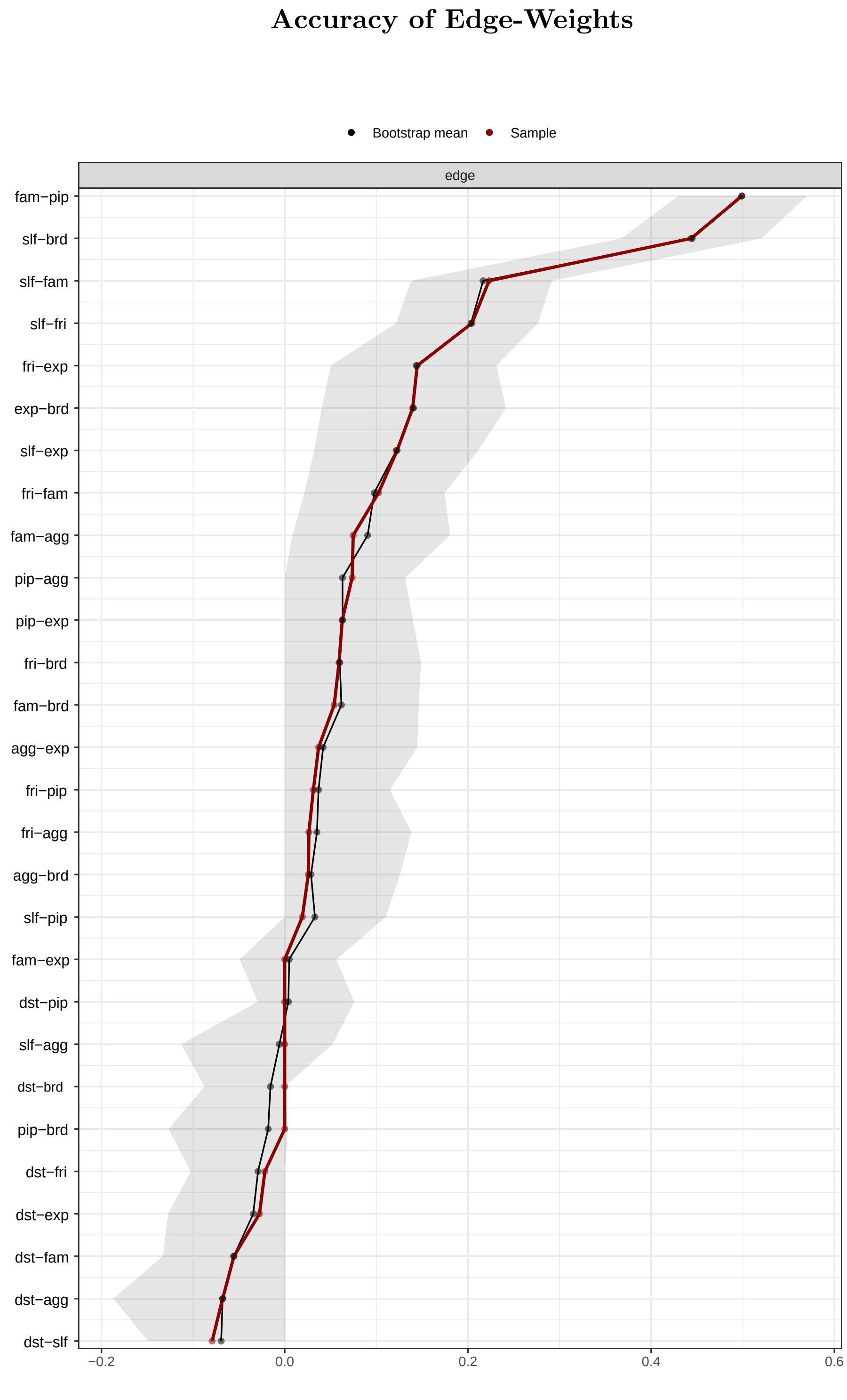

Figure 10: Accuracy of edge-weights and their non-parametric bootstrapped 95% confidence intervals for the lasso regularised network including pre-pandemic resilience factors and pandemic-related distress responses as measured by the K6 extended residuals. Edges of the network are displayed on the y-axis, ordered from lowest (bottom) to highest (top) edge (for a legend of variable labels, see Figure 8 or Figure 9). The grey area around the red (observed sample) and black (bootstrapped means) dots indicates the confidence interval; the more reliable an edge, the smaller its confidence interval.

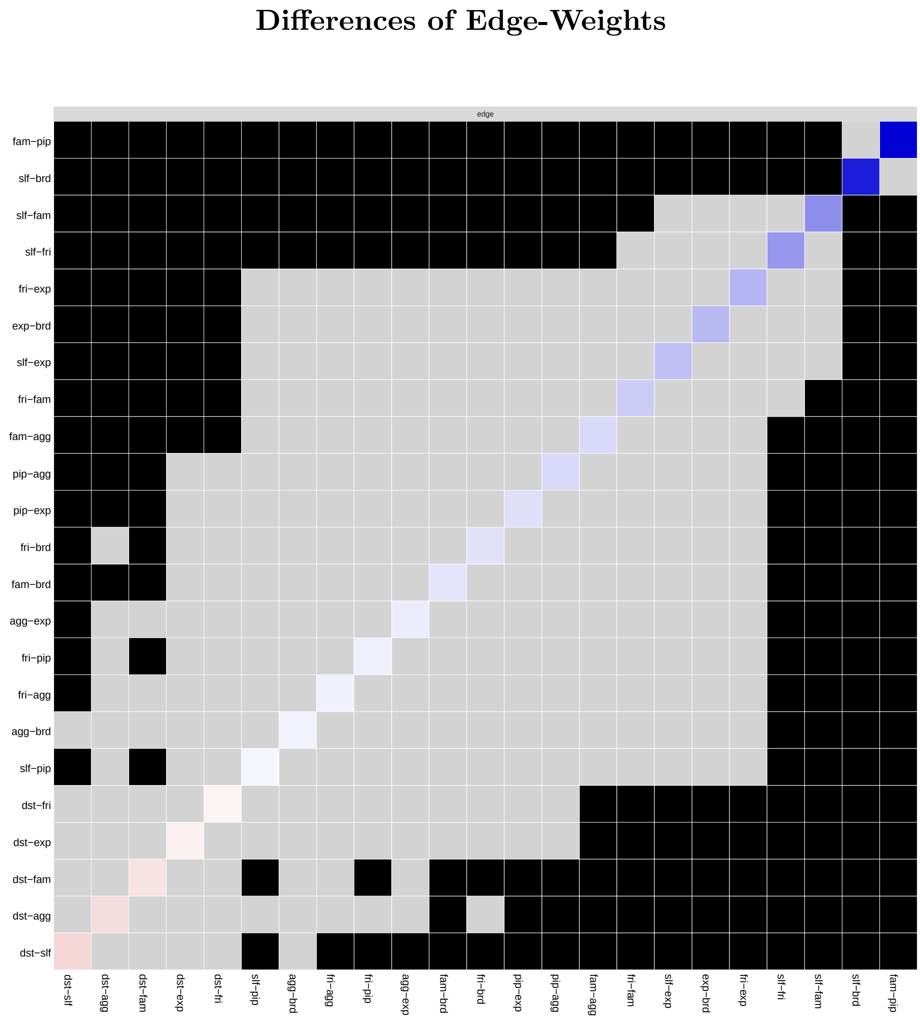

Figure 11: Differences of edge-weights for the lasso regularised network including pre-pandemic resilience factors and pandemic-related distress responses as measured by the K6 extended residuals. Edges are listed on both the x- and y-axis (for a legend of variable labels, see Figure 8 or Figure 9); *grey* boxes indicate edges that are not significantly different from each other whilst *black* boxes indicate edges that are significantly different from each other (significance level $\alpha = 0.05$; please note that the test does not control for multiple comparisons). The diagonally coloured boxes refer to the edge colour of the network graph where positive relations are blue and negative relations are red.

Summary No. 2:

As can be seen in Figure 10, the confidence intervals for many of the estimated edge-weights overlap, suggesting that these edge-weights likely do not significantly differ from each other which, in turn, is confirmed by the edge-weight difference test depicted in Figure 11. Whilst the confidence intervals are sizeable in some cases (indicating that their order should be interpreted with care), there is little overlap between the intervals of negatively and positively weighted edges, indicating that the sign of these effects is robust within the data.

Centrality Indices

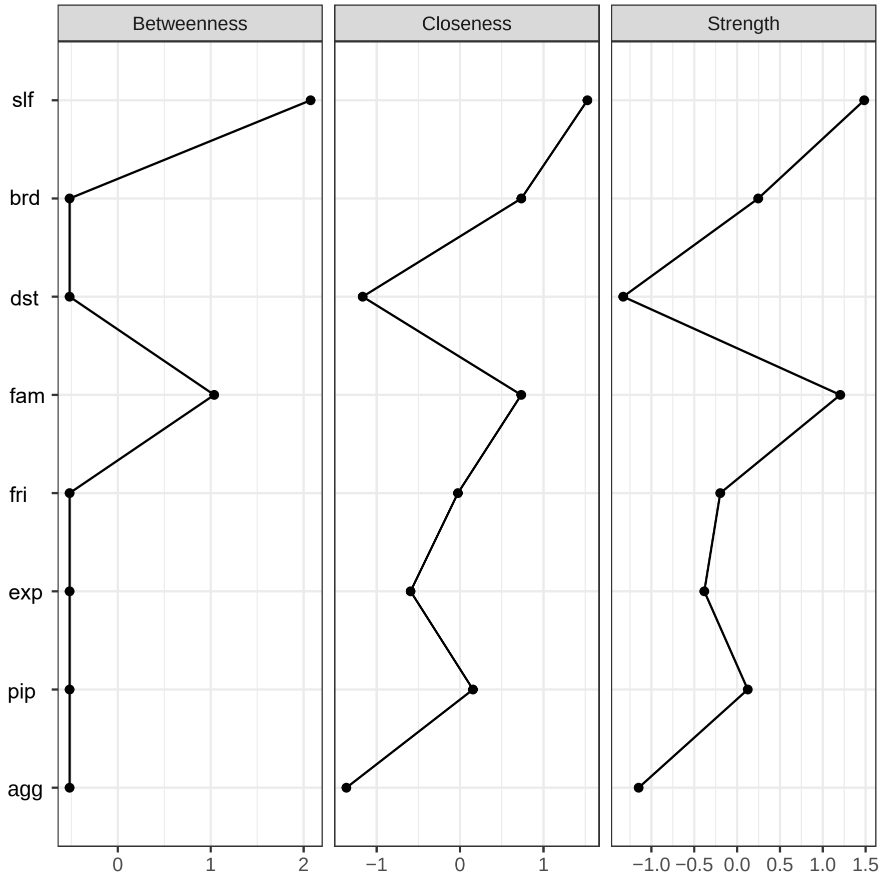

Figure 12: Centrality indices shown as standardised z-scores for the lasso regularised network including pre-pandemic resilience factors and pandemic-related distress responses as measured by the K6 extended residuals; for a legend of variable labels, see Figure 8 or Figure 9; for standardised raw scores, see Table 13; for centrality stability, see Figure 13.

Table 13: Standardised centrality indices.

	dst	slf	fri	fam	pip	agg	exp	brd
betweenness	-0.52	2.08	-0.52	1.04	-0.52	-0.52	-0.52	-0.52
closeness	-1.17	1.53	-0.03	0.73	0.15	-1.36	-0.59	0.73
strength	-1.33	1.48	-0.20	1.20	0.12	-1.15	-0.38	0.25

Note: For a legend of variable labels, see Figure 8 or Figure 9.

Stability of Centrality Indices

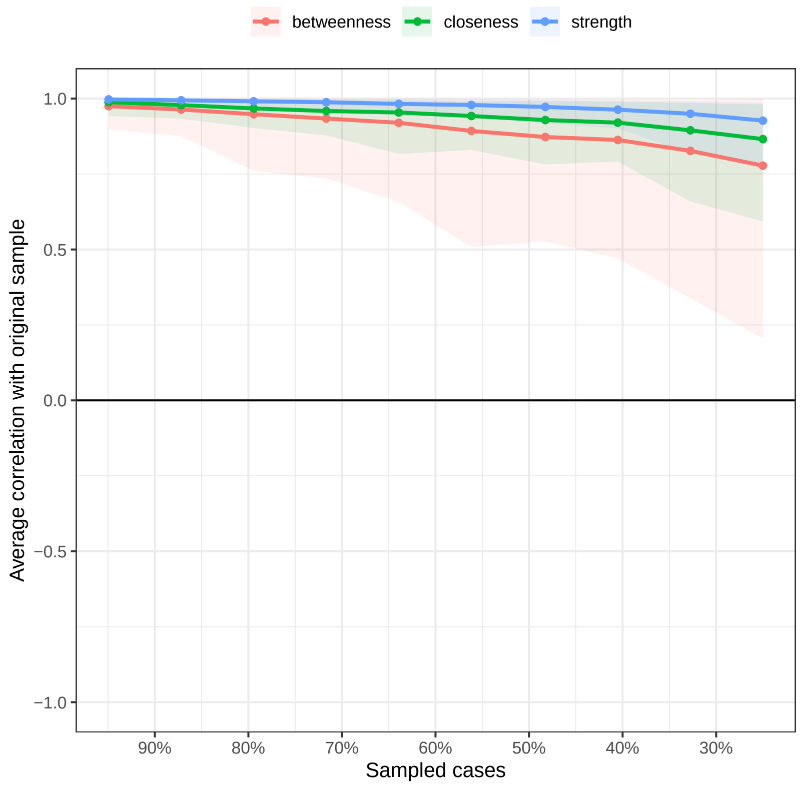

Figure 13: Stability of centrality indices; CS-coefficient for betweenness ($CS(\text{cor} = 0.7) = 0.44$), closeness ($CS(\text{cor} = 0.7) = 0.52$), and strength ($CS(\text{cor} = 0.7) = 0.75$) indicate that betweenness is unstable and should be disregarded, closeness should be interpreted with care, however, strength indices are stable.

Summary No. 3:

Centrality indices show that some nodes differ quite substantially in their estimates. Self-esteem, for instance, has the highest betweenness, closeness, and strength. Using case-dropping subset bootstrap, we can further examine their stability, otherwise they would be difficult to interpret. Figure 13 shows that node strength seems to be the most stable estimate of the three assessed centrality indices. This is confirmed by the *correlation stability coefficient* (CS-coefficient). The CS-coefficient expresses the highest proportion of cases which can be discarded whilst retaining a correlation to the measured centrality above a given threshold, here using the default of 0.7, with 95% confidence. Preferably, it should be above 0.5.[26] Node betweenness ($CS(\text{cor} = 0.7) = 0.44$) was below this threshold, node closeness ($CS(\text{cor} = 0.7) = 0.52$) only just met this relatively arbitrary cut-off and should be interpreted with care, node strength ($CS(\text{cor} = 0.7) = 0.75$), however, was high, suggesting sufficient stability.

Supplementary Materials 6b

Further Network Analysis Results (SWEMWBS)

Association Network

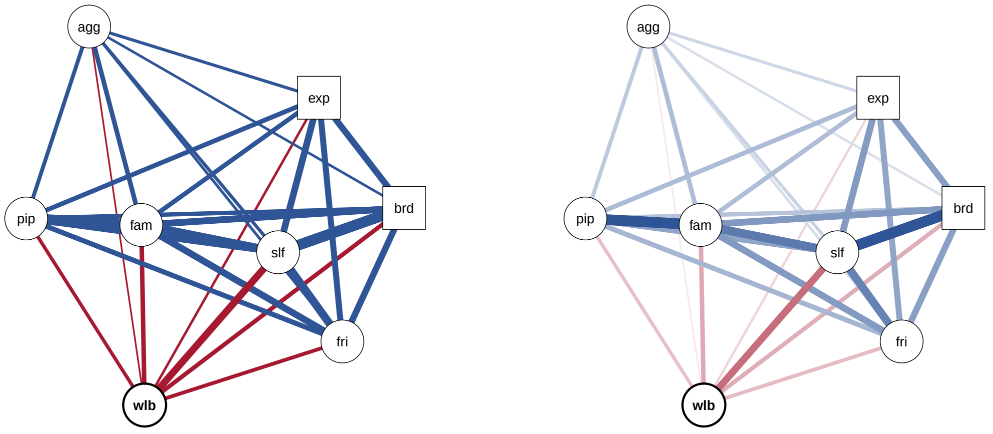

(a) Unfaded Association Network

(b) Faded Association Network

Figure 14: Association network including pre-pandemic resilience factors and pandemic-related mental wellbeing response measured by SWEMWBS extended residual scores where lower scores indicate lower-than-expected mental wellbeing during the first national lockdown compared with expected levels over seven years (n = 620). All resilience factors are coded so that higher scores indicate higher protection. **Legend**: pandemic-related mental wellbeing response/SWEMWBS extended residuals [**wlb**], self-esteem [**slf**], friendship support [**fri**], general family functioning [**fam**], positive and involved parenting [**pip**], low aggression [**agg**], low expressive suppresion [**exp**], low ruminative brooding [**brd**]. Nodes surrounded by a circle denote factor scores, nodes surrounded by a square denote single item-level (categorical) scores. Edge colour refers to either positive (blue) or negative (red) relations.

Table 14: Corresonding correlation matrix to Figure 14.

	wlb	slf	fri	fam	pip	agg	exp	brd
wlb	1.00							
slf	-0.38	1.00						
fri	-0.18	0.44	1.00					
fam	-0.23	0.47	0.36	1.00				
pip	-0.17	0.32	0.26	0.61	1.00			
agg	-0.06	0.16	0.12	0.24	0.19	1.00		
exp	-0.11	0.36	0.32	0.23	0.24	0.14	1.00	
brd	-0.21	0.61	0.34	0.37	0.21	0.11	0.34	1.00

Note: We used the *cor_auto* function of the R-package *qgraph* to automatically compute the appropriate correlation matrix based on polychoric and Pearson correlations. For a legend, see Figure 14.

Lasso Regularised Network

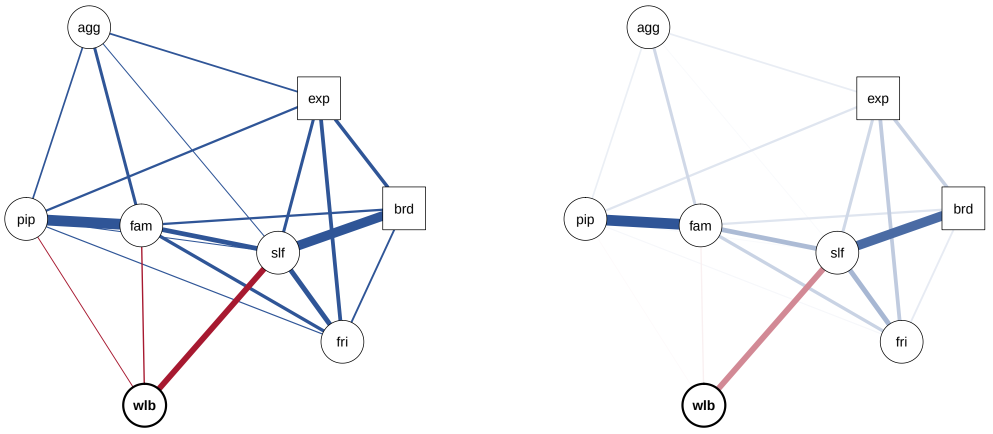

(a) Unfaded Lasso Regularised Network

(b) Faded Lasso Regularised Network

Figure 15: Lasso regularised network including pre-pandemic resilience factors and pandemic-related mental wellbeing response measured by SWEMWBS extended residual scores where lower scores indicate lower-than-expected mental wellbeing during the first national lockdown compared with expected levels over seven years ($n = 620$). All resilience factors are coded so that higher scores indicate higher protection. **Legend**: pandemic-related distress response/SWEMWBS extended residuals [**wlb**], self-esteem [**slf**], friendship support [**fri**], general family functioning [**fam**], positive and involved parenting [**pip**], low aggression [**agg**], low expressive suppression [**exp**], low ruminative brooding [**brd**]. Nodes surrounded by a circle denote factor scores, nodes surrounded by a square denote single item-level (categorical) scores. Edge colour refers to either positive (blue) or negative (red) relations.

Table 15: Corresponding weights matrix for Figure 15.

	wlb	slf	fri	fam	pip	agg	exp	brd
wlb	0.00							
slf	-0.25	0.00						
fri	0.00	0.21	0.00					
fam	-0.03	0.20	0.13	0.00				
pip	-0.01	0.01	0.02	0.49	0.00			
agg	0.00	0.01	0.00	0.11	0.05	0.00		
exp	0.00	0.11	0.15	0.00	0.07	0.05	0.00	
brd	0.00	0.43	0.06	0.08	0.00	0.00	0.13	0.00

Note: For a legend, see Figure 15.

Summary No. 1:

Similar to what has been discussed in Supplementary Materials 6a, the association network in Figure 14 shows that all resilience factors are positively (blue edges), and mostly strongly (edge thickness), correlated. The negative correlations (red edges) between resilience factors and pandemic-related mental wellbeing response as measured by the SWEMWBS extended residuals mean that lower-than-expected mental wellbeing was related to higher resilience scores and vice versa. The lasso regularised network model is discussed in detail within the main article.

Accuracy of Edge-Weights

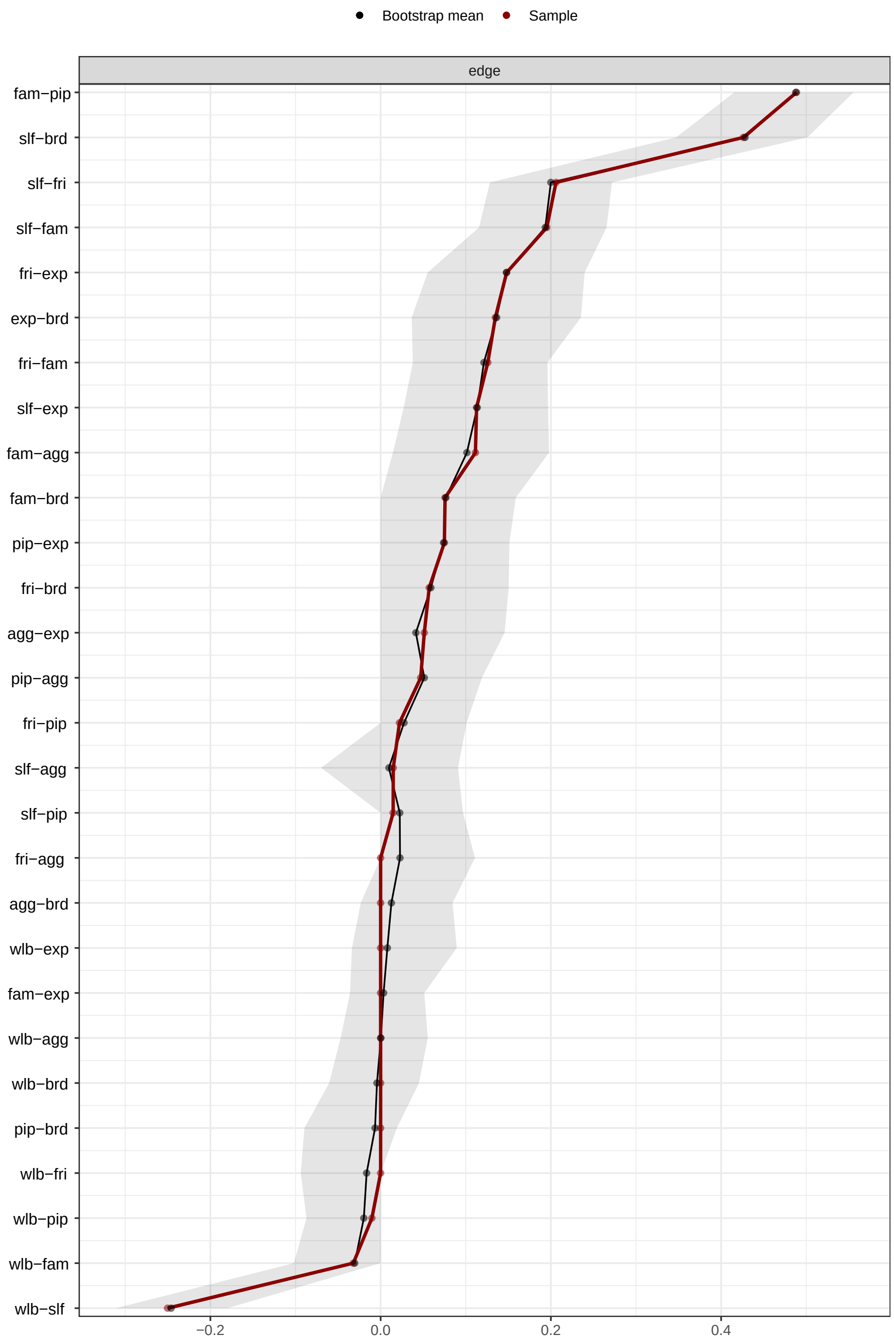

Figure 16: Accuracy of edge-weights and their non-parametric bootstrapped 95% confidence intervals for the lasso regularised network including pre-pandemic resilience factors and pandemic-related mental wellbeing responses as measured by the SWEMWBS extended residuals. Edges of the network are displayed on the y-axis, ordered from lowest (bottom) to highest (top) edge (for a legend of variable labels, see Figure 14 or Figure 15). The grey area around the red (observed sample) and black (bootstrapped means) dots indicates the confidence interval; the more reliable an edge, the smaller its confidence interval.

Differences of Edge-Weights

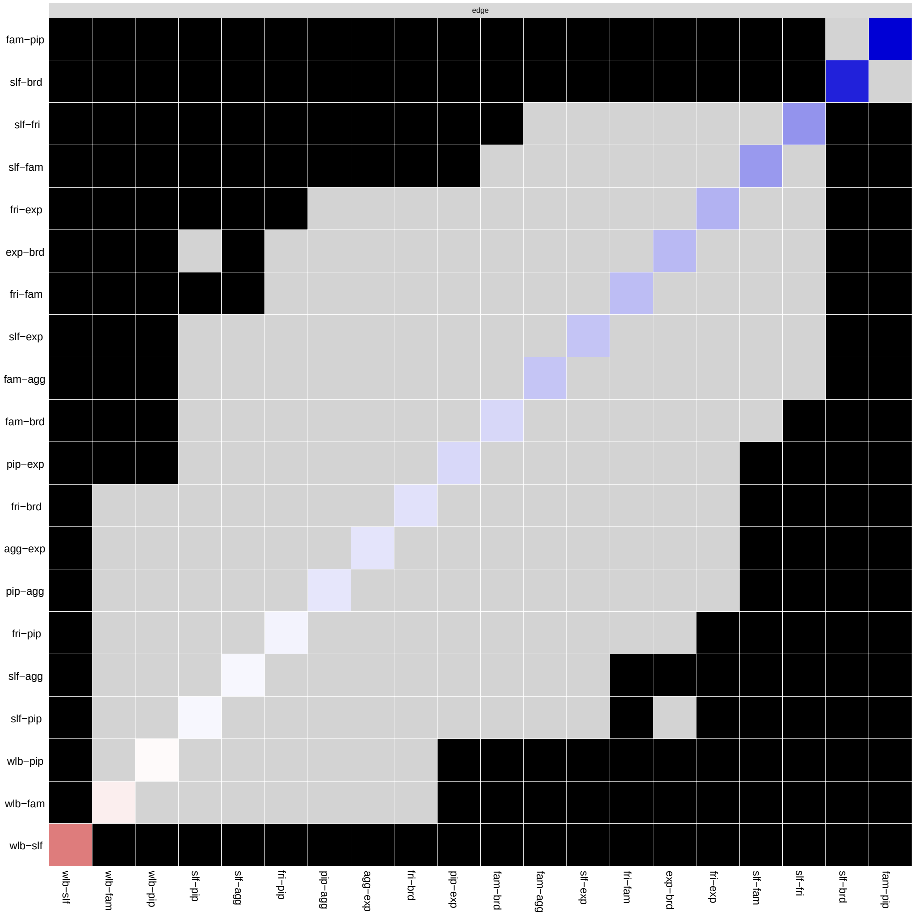

Figure 17: Differences of edge-weights for the lasso regularised network including pre-pandemic resilience factors and pandemic-related mental wellbeing responses as measured by the SWEMWBS extended residuals. Edges are listed on both the x- and y-axis (for a legend of variable labels, see Figure 14 or Figure 15); *grey* boxes indicate edges that are not significantly different from each other whilst *black* boxes indicate edges that are significantly different from each other (significance level $\alpha = 0.05$; please note that the test does not control for multiple comparisons). The diagonally coloured boxes refer to the edge colour of the network graph where positive relations are blue and negative relations are red.

Summary No. 2:

Similar to what has been discussed in Supplementary Materials 6a, the confidence intervals for many of the estimated edge-weights overlap (see Figure 16) This suggests that these edge-weights likely do not significantly differ from each other which, in turn, is confirmed by the edge-weight difference test depicted in Figure 17. Further it can be seen that the edge between self-esteem (slf) and the extended residual (wlb) differs significantly from any other edge, hence, showing high accuracy, particularly when compared to the other two edges connecting the extended residual, *i.e.*, family functioning (fam) and positive and involved parenting (pip).

Centrality Indices

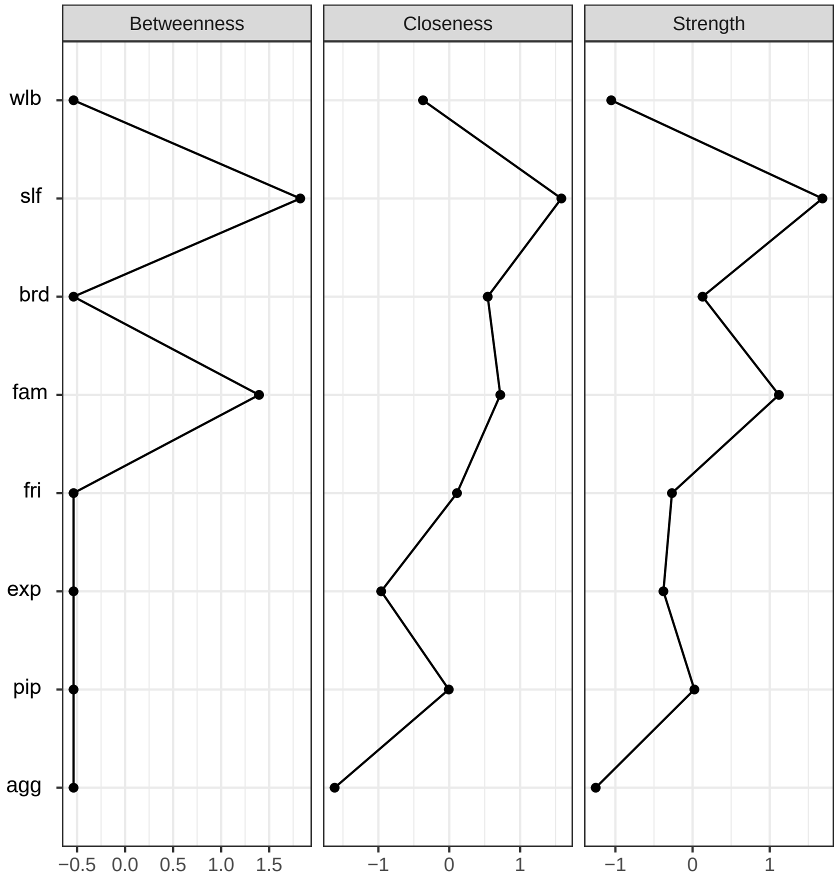

Figure 18: Centrality indices shown as standardised z-scores for the lasso regularised network including pre-pandemic resilience factors and pandemic-related mental wellbeing responses as measured by the SWEMWBS extended residuals; for a legend of variable labels, see Figure 14 or Figure 15; for standardised raw scores, see Table 16; for centrality stability, see Figure 19.

Table 16: Standardised centrality indices.

	wlb	slf	fri	fam	pip	agg	exp	brd
betweenness	-0.54	1.82	-0.54	1.39	-0.54	-0.54	-0.54	-0.54
closeness	-0.37	1.58	0.11	0.72	-0.01	-1.62	-0.96	0.54
strength	-1.05	1.68	-0.27	1.12	0.02	-1.25	-0.38	0.13

Note: For a legend of variable labels, see Figure 14 or Figure 15.

Stability of Centrality Indices

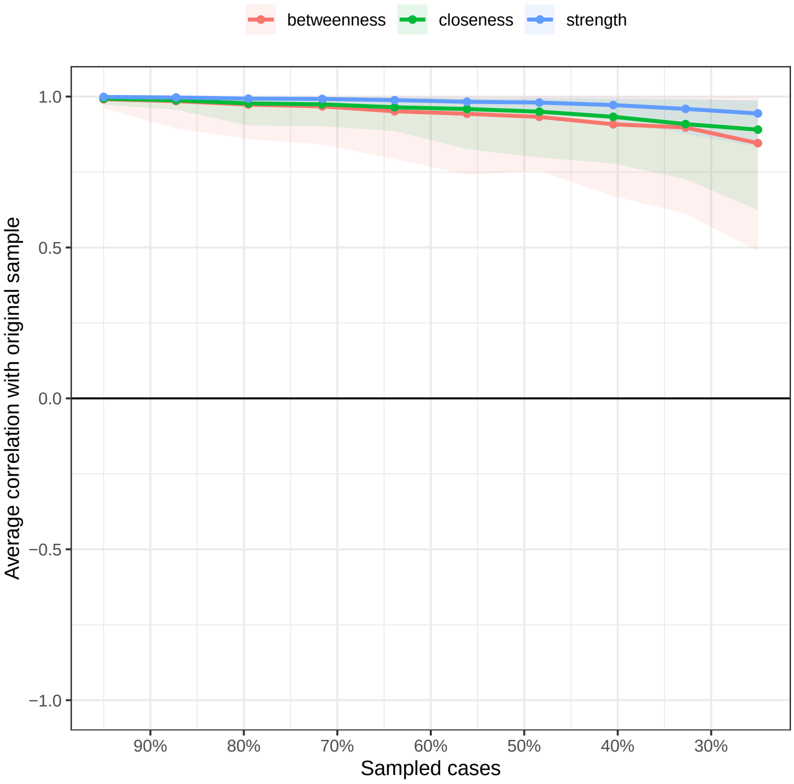

Figure 19: Stability of centrality indices; CS-coefficient for betweenness ($CS(\text{cor} = 0.7) = 0.67$), closeness ($CS(\text{cor} = 0.7) = 0.60$), and strength ($CS(\text{cor} = 0.7) = 0.75$) indicate that indices are stable.

Summary No. 3:

Centrality indices show that some nodes differ quite substantially in their estimates. Again, similar to what has been discussed in Supplementary Materials 6a, self-esteem, for instance, has the highest betweenness, closeness, and strength. Using case-dropping subset bootstrap, we further examined their stability. Figure 19 shows that node strength seems to be the most stable estimate of the three assessed centrality indices, however, both betweenness as well as closeness appear relatively stable as well. This is confirmed by the *correlation stability coefficient* (CS-coefficient; see Supplementary Materials 6a, Summary No. 3 for explanation). Node betweenness ($CS(\text{cor} = 0.7) = 0.67$), node closeness ($CS(\text{cor} = 0.7) = 0.60$) as well as node strength ($CS(\text{cor} = 0.7) = 0.75$) all show sufficient stability ($CS(\text{cor} = 0.7) > 0.5$).[26]

Supplementary Materials 7a

Further Correlational Analysis (K6)

Observed K6 Association Network

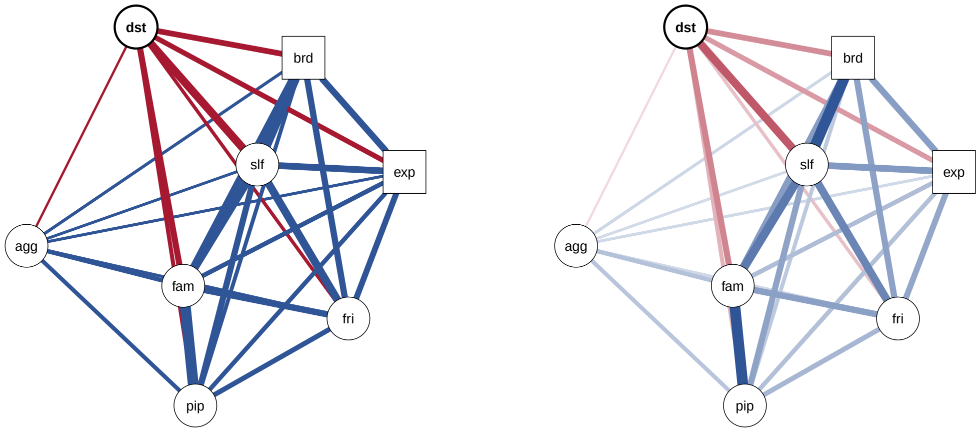

(a) Unfaded Association Network **(b)** Faded Association Network

Figure 20: Association network including pre-pandemic resilience factors and observed psychological distress measured by the K6 where higher scores indicate higher psychological distress during the first national lockdown (n = 632). All resilience factors are coded so that higher scores indicate higher protection. **Legend**: psychological distress/K6 [**dst**], self-esteem [**slf**], friendship support [**fri**], general family functioning [**fam**], positive and involved parenting [**pip**], low aggression [**agg**], low expressive suppression [**exp**], low ruminative brooding [**brd**]. Nodes surrounded by a circle denote factor scores, nodes surrounded by a square denote single item-level (categorical) scores. Edge colour refers to either positive (blue) or negative (red) relations. Note that within the faded network the colour fades the weaker the edge weight is.

Table 17: Corresponding correlation matrix to Figure 20.

	dst	slf	fri	fam	pip	agg	exp	brd
dst	1.00							
slf	-0.45	1.00						
fri	-0.17	0.44	1.00					
fam	-0.33	0.48	0.34	1.00				
pip	-0.20	0.33	0.26	0.61	1.00			
agg	-0.10	0.13	0.14	0.22	0.21	1.00		
exp	-0.27	0.37	0.32	0.24	0.23	0.14	1.00	
brd	-0.30	0.61	0.34	0.35	0.20	0.15	0.35	1.00

Note: We used the *cor_auto* function of the R-package *qgraph* to automatically compute the appropriate correlation matrix based on polychoric and Pearson correlations. For a legend, see Figure 20.

Supplementary Materials 7b

Further Correlational Analysis (SWEMWBS)

Observed SWEMWBS Association Network

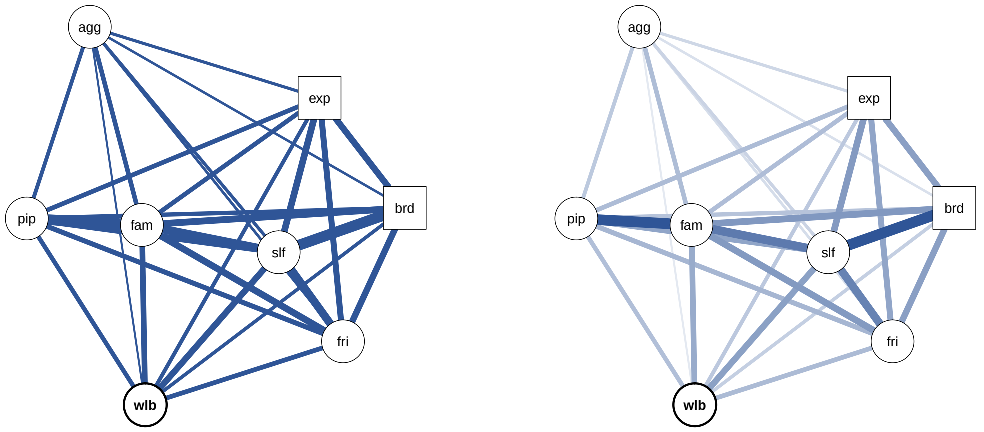

(a) Unfaded Association Network **(b)** Faded Association Network

Figure 21: Association network including pre-pandemic resilience factors and observed mental wellbeing measured by the SWEMWBS where higher scores indicate better mental wellbeing during the first national lockdown ($n = 620$). All resilience factors are coded so that higher scores indicate higher protection. **Legend**: mental wellbeing/SWEMWBS [**wlb**], self-esteem [**slf**], friendship support [**fri**], general family functioning [**fam**], positive and involved parenting [**pip**], low aggression [**agg**], low expressive suppression [**exp**], low ruminative brooding [**brd**]. Nodes surrounded by a circle denote factor scores, nodes surrounded by a square denote single item-level (categorical) scores. Edge colour refers to either positive (blue) or negative (red) relations. Note that within the faded network the colour fades the weaker the edge weight is.

Table 18: Corresponding correlation matrix to Figure 21.

	wlb	slf	fri	fam	pip	agg	exp	brd
wlb	1.00							
slf	0.34	1.00						
fri	0.24	0.44	1.00					
fam	0.30	0.47	0.36	1.00				
pip	0.23	0.32	0.26	0.60	1.00			
agg	0.08	0.16	0.12	0.24	0.19	1.00		
exp	0.20	0.36	0.32	0.23	0.24	0.14	1.00	
brd	0.17	0.61	0.34	0.37	0.21	0.11	0.34	1.00

Note: We used the *cor_auto* function of the R-package *qgraph* to automatically compute the appropriate correlation matrix based on polychoric and Pearson correlations. For a legend, see Figure 21.

References

[1] Michael N. Hallquist and Joshua F. Wiley. MplusAutomation: An R package for facilitating large-scale latent variable analyses in Mplus. *Structural Equation Modeling*, pages 1–18, 2018.

[2] Linda K. Muthén and Muthén Bengt O. MPLUS user's guide. *https://www.statmodel.com/*, 1998-2017.

[3] Litze Hu and Peter M Bentler. Cutoff criteria for fit indexes in covariance structure analysis: Conventional criteria versus new alternatives. *Structural Equation Modeling: A Multidisciplinary Journal*, 6(1):1–55, 1999.

[4] Subhash Sharma, Soumen Mukherjee, Ajith Kumar, and William R Dillon. A simulation study to investigate the use of cutoff values for assessing model fit in covariance structure models. *Journal of Business Research*, 58(7):935–943, 2005.

[5] Dennis L Jackson, J Arthur Gillaspy Jr, and Rebecca Purc-Stephenson. Reporting practices in confirmatory factor analysis: An overview and some recommendations. *Psychological Methods*, 14(1):6, 2009.

[6] Yan Xia and Yanyun Yang. Rmsea, cfi, and tli in structural equation modeling with ordered categorical data: The story they tell depends on the estimation methods. *Behavior Research Methods*, 51(1):409–428, 2019.

[7] Jessica Fritz, Anne M de Graaff, Helen Caisley, Anne-Laura Van Harmelen, and Paul O Wilkinson. A systematic review of amenable resilience factors that moderate and/or mediate the relationship between childhood adversity and mental health in young people. *Frontiers in Psychiatry*, 9:230, 2018.

[8] Jessica Fritz, Eiko I Fried, Ian M Goodyer, Paul O Wilkinson, and A-L van Harmelen. A network model of resilience factors for adolescents with and without exposure to childhood adversity. *Scientific Reports*, 8(1):1–13, 2018.

[9] Jessica Fritz, Jan Stochl, Eiko I Fried, Ian M Goodyer, Claudia D van Borkulo, Paul O Wilkinson, and A-L Van Harmelen. Unravelling the complex nature of resilience factors and their changes between early and later adolescence. *BMC Medicine*, 17(1):1–16, 2019.

[10] Jessica Fritz, Jan Stochl, Ian M Goodyer, A-L van Harmelen, and Paul O Wilkinson. Embracing the positive: An examination of how well resilience factors at age 14 can predict distress at age 17. *Translational Psychiatry*, 10(1):1–14, 2020.

[11] Paul J Frick, Rachel E Christian, and Jane M Wootton. Age trends in the association between parenting practices and conduct problems. *Behavior Modification*, 23(1):106–128, 1999.

[12] Frank J Elgar, Daniel A Waschbusch, Mark R Dadds, and Nadine Sigvaldason. Development and validation of a short form of the Alabama Parenting Questionnaire. *Journal of Child and Family Studies*, 16(2):243–259, 2007.

[13] Kimberly R Zlomke, Dustin Lamport, Sarah Bauman, Beth Garland, and Brett Talbot. Parenting adolescents: Examining the factor structure of the alabama parenting questionnaire for adolescents. *Journal of Child and Family Studies*, 23(8):1484–1490, 2014.

[14] Ian M Goodyer, Sonya Tsancheva, Sarah Byford, Bernadka Dubicka, Jonathan Hill, Raphael Kelvin, Shirley Reynolds, Christopher Roberts, Robert Senior, John Suckling, et al. Improving mood with psychoanalytic and cognitive therapies (impact): a pragmatic effectiveness superiority trial to investigate whether specialised psychological treatment reduces the risk for relapse in adolescents with moderate to severe unipolar depression: Study protocol for a randomised controlled trial. *Trials*, 12(1):1–12, 2011.

[15] IM Goodyer, C Wright, and PME Altham. Recent friendships in anxious and depressed school age children. *Psychological Medicine*, 19(1):165–174, 1989.

[16] Nathan B Epstein, Lawrence M Baldwin, and Duane S Bishop. The mcmaster family assessment device. *Journal of Marital and Family Therapy*, 9(2):171–180, 1983.

[17] Morris Rosenberg. Self esteem and the adolescent. *Science*, 148(3671):804, 1965.

[18] Laura Salerno, Sonia Ingoglia, and Gianluca Lo Coco. Competing factor structures of the rosenberg self-esteem scale (rses) and its measurement invariance across clinical and non-clinical samples. *Personality and Individual Differences*, 113:13–19, 2017.

[19] Norman G Poythress, Kevin S Douglas, Diana Falkenbach, Keith Cruise, Zina Lee, Daniel C Murrie, and Michael Vitacco. Internal consistency reliability of the self-report antisocial process screening device. *Assessment*, 13(1):107–113, 2006.

[20] Diane Bamber, Alison Tamplin, Rebecca J Park, Zoe A Kyte, and Ian M Goodyer. Development of a short leyton obsessional inventory for children and adolescents. *Journal of the American Academy of Child & Adolescent Psychiatry*, 41(10):1246–1252, 2002.

[21] Sture Holm. A simple sequentially rejective multiple test procedure. *Scandinavian Journal of Statistics*, 6(2):65–70, 1979.

[22] Sacha Epskamp, Angélique O. J. Cramer, Lourens J. Waldorp, Verena D. Schmittmann, and Denny Borsboom. qgraph: Network visualizations of relationships in psychometric data. *Journal of Statistical Software*, 48(4):1–18, 2012.

[23] Rina Foygel and Mathias Drton. Extended bayesian information criteria for gaussian graphical models. *arXiv preprint arXiv:1011.6640*, 2010.

[24] Jerome Friedman, Trevor Hastie, and Robert Tibshirani. Sparse inverse covariance estimation with the graphical lasso. *Biostatistics*, 9(3):432–441, 2008.

[25] Laura F Bringmann, Timon Elmer, Sacha Epskamp, Robert W Krause, David Schoch, Marieke Wichers, Johanna TW Wigman, and Evelien Snippe. What do centrality measures measure in psychological networks? *Journal of Abnormal Psychology*, 128(8):892, 2019.

[26] Sacha Epskamp, Denny Borsboom, and Eiko I. Fried. Estimating psychological networks and their accuracy: A tutorial paper. *Behavior Research Methods*, 2017.

[27] Sacha Epskamp and Eiko I Fried. A tutorial on regularized partial correlation networks. *Psychological Methods*, 23(4):617, 2018.
